# Supplementary material for: Spatially visualized single-cell pathology of highly multiplexed protein profiles in health and disease
Source: Commun Biol. 2021 May 27;4:632. doi: 10.1038/s42003-021-02166-2 (PMC8160218; doi:10.1038/s42003-021-02166-2)
Supplement: Supplementary file 2 — Supplementary Information [file 42003_2021_2166_MOESM2_ESM.pdf]

Supplementary Information

Spatially visualized single-cell pathology of highly multiplexed protein profiles in health and disease

Mayar Allam[1,#], Thomas Hu[1,2,#], Shuangyi Cai[1], Krishnan Laxminarayanan[3], Robert B. Hughley[3], and Ahmet F. Coskun[1,3,4*]

[1] Wallace H. Coulter Department of Biomedical Engineering, Georgia Institute of Technology and Emory University, Atlanta, GA, USA.

[2] School of Electrical and Computer Engineering, Georgia Institute of Technology, Atlanta, GA, USA.

[3] Parker H. Petit Institute for Bioengineering and Bioscience, Georgia Institute of Technology, 315 Ferst Dr. NW, Atlanta, GA 30332

[4] Interdisciplinary Bioengineering Graduate Program, Georgia Institute of Technology, Atlanta, GA, USA.

[#]These authors contributed equally *Corresponding author

Ahmet F. Coskun, Ph.D. (ahmet.coskun@bme.gatech.edu)

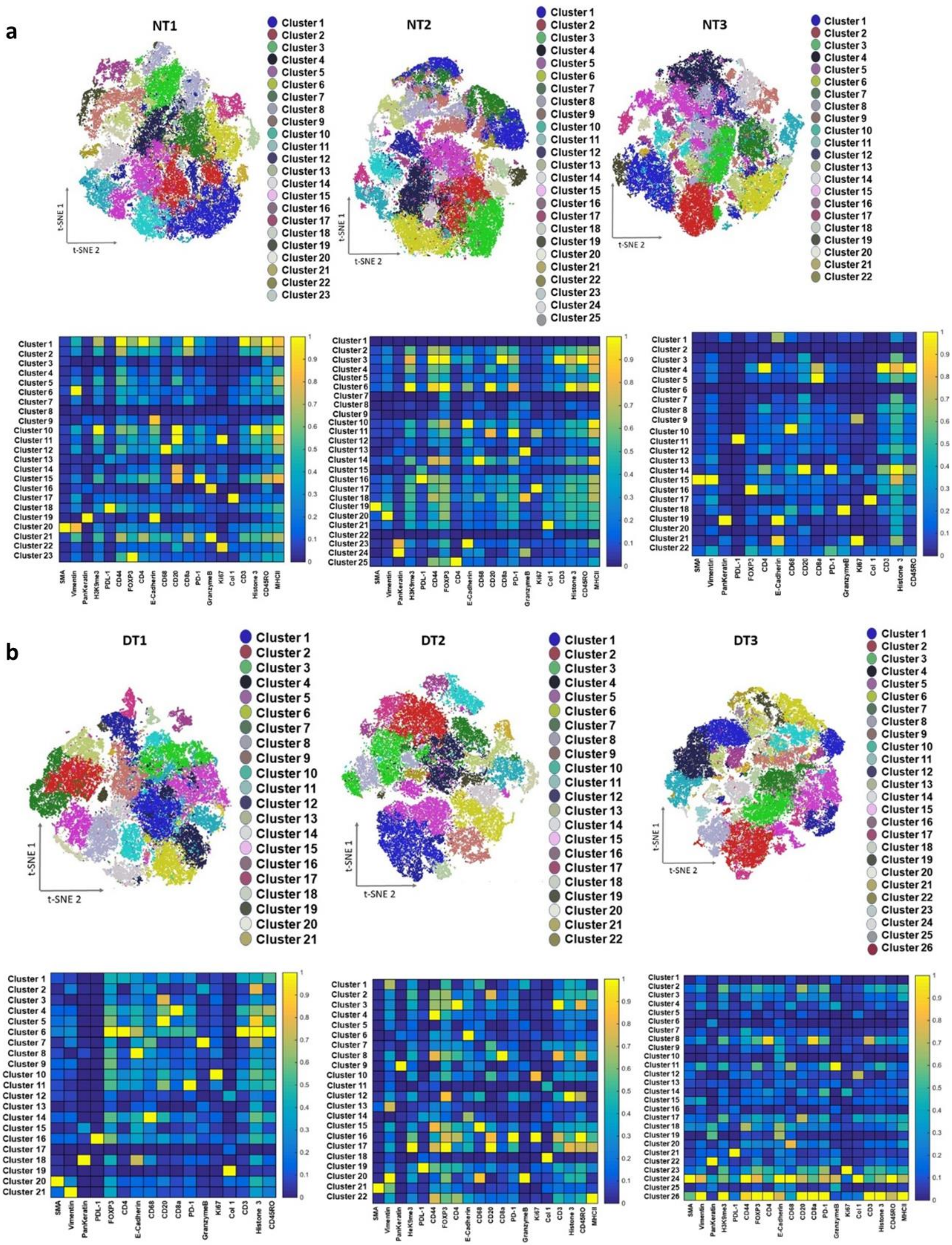
a
NT1
NT2
NT3
b
DT1
DT2
DT3
t-SNE 1
t-SNE 2
Cluster 1
Cluster 2
Cluster 3
Cluster 4
Cluster 5
Cluster 6
Cluster 7
Cluster 8
Cluster 9
Cluster 10
Cluster 11
Cluster 12
Cluster 13
Cluster 14
Cluster 15
Cluster 16
Cluster 17
Cluster 18
Cluster 19
Cluster 20
Cluster 21
Cluster 22
Cluster 23
Cluster 24
Cluster 25
Cluster 26
SMA
Vimentin
PanKeratin
H3K9me3
PDL-1
FOXP3
CD44
CD4
E-Cadherin
CD68
CD20
CD8a
PD-1
GranzymeB
Ki67
Col 1
CD3
Histone 3
CD45RO
MHCII
0
0.1
0.2
0.3
0.4
0.5
0.6
0.7
0.8
0.9
1

Supplementary Figure 1. PhenoGraphs of normal and diseased tonsils reveal the heterogeneity of markers distribution.

a PhenoGraphs of all three normal ROIs showed all clusters' distribution and associated heatmaps that exhibited marker expressions in each cluster.

b PhenoGraphs of all three diseased ROIs demonstrated the distribution of all clusters and their related heatmaps that showed the marker expressions in each cluster.

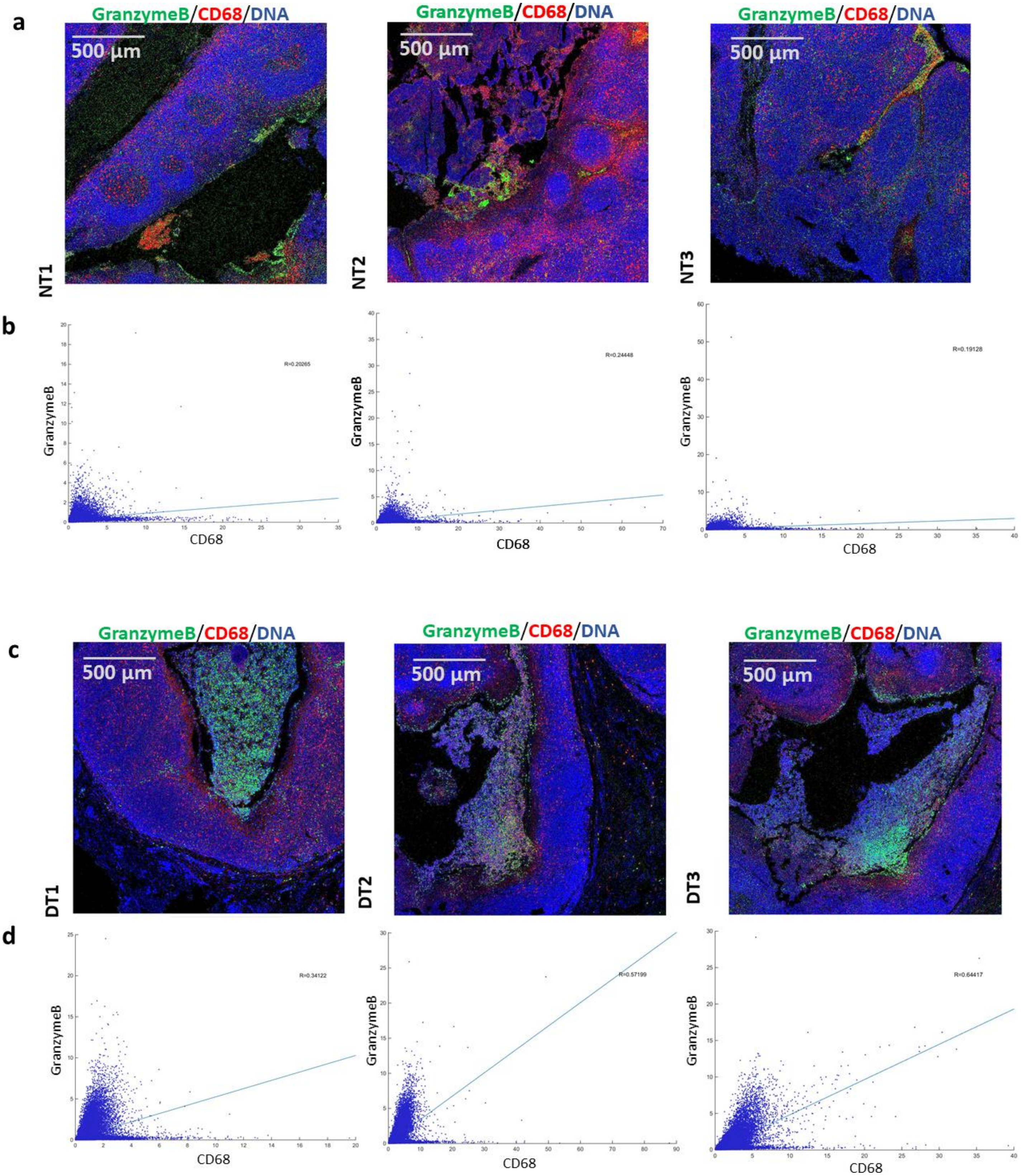
a
GranzymeB/CD68/DNA
500 µm
NT1
NT2
NT3
b
GranzymeB
CD68
R=0.20265
R=0.24448
R=0.19128
c
DT1
DT2
DT3
d
R=0.34122
R=0.57199
R=0.64417

Supplementary Figure 2. CD68+ cells are associated with Granzyme B expression in response to infection in the diseased tonsil.

a Visual representation showed the co-expression of granzyme B and CD68 across three normal tonsils ROIs.

b Correlation analysis between granzyme B and CD68 at the single-cell level across three normal tonsils ROIs was presented. Pearson's correlation coefficient was found to be R = 0.20265 for NT1, R = 0.24448 for NT2, and 0.19128 for NT3.

c Visual representation demonstrated the co-expression of granzyme B and CD68 across three diseased tonsils ROIs.

d Correlation analysis between granzyme B and CD68 at the single-cell level across three diseased tonsils ROIs was shown. Pearson's correlation coefficient was found to be R = 0.34122 for DT1, R = 0.57199 for DT2, and 0.64417 for DT3.

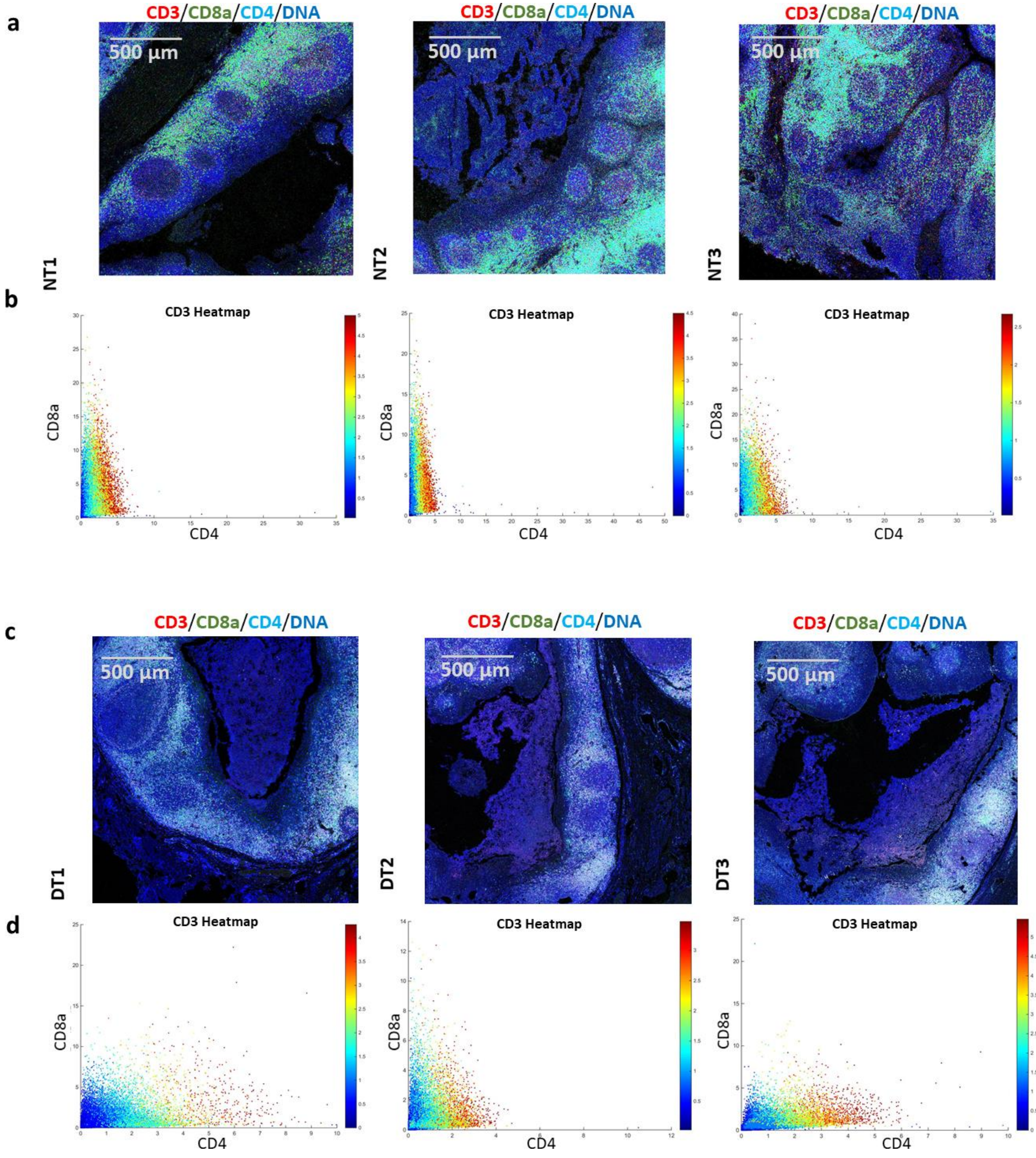
a
CD3/CD8a/CD4/DNA
500 µm
NT1
NT2
NT3
b
CD3 Heatmap
CD8a
CD4
c
DT1
DT2
DT3
d

Supplementary Figure 3. CD3+ cells preferentially co-express CD4 in response to infection

a Visual representation showed the co-expression of CD3, CD4, CD8a across three normal tonsils ROIs.

b Correlation analysis between CD4 and CD8a with CD3 heatmap across three normal tonsils ROIs was demonstrated.

c Visual representation provided the co-expression of CD3, CD4, CD8a across three diseased tonsils ROIs.

d Correlation analysis between CD4 and CD8a with CD3 heatmap across three diseased tonsils ROIs was provided.

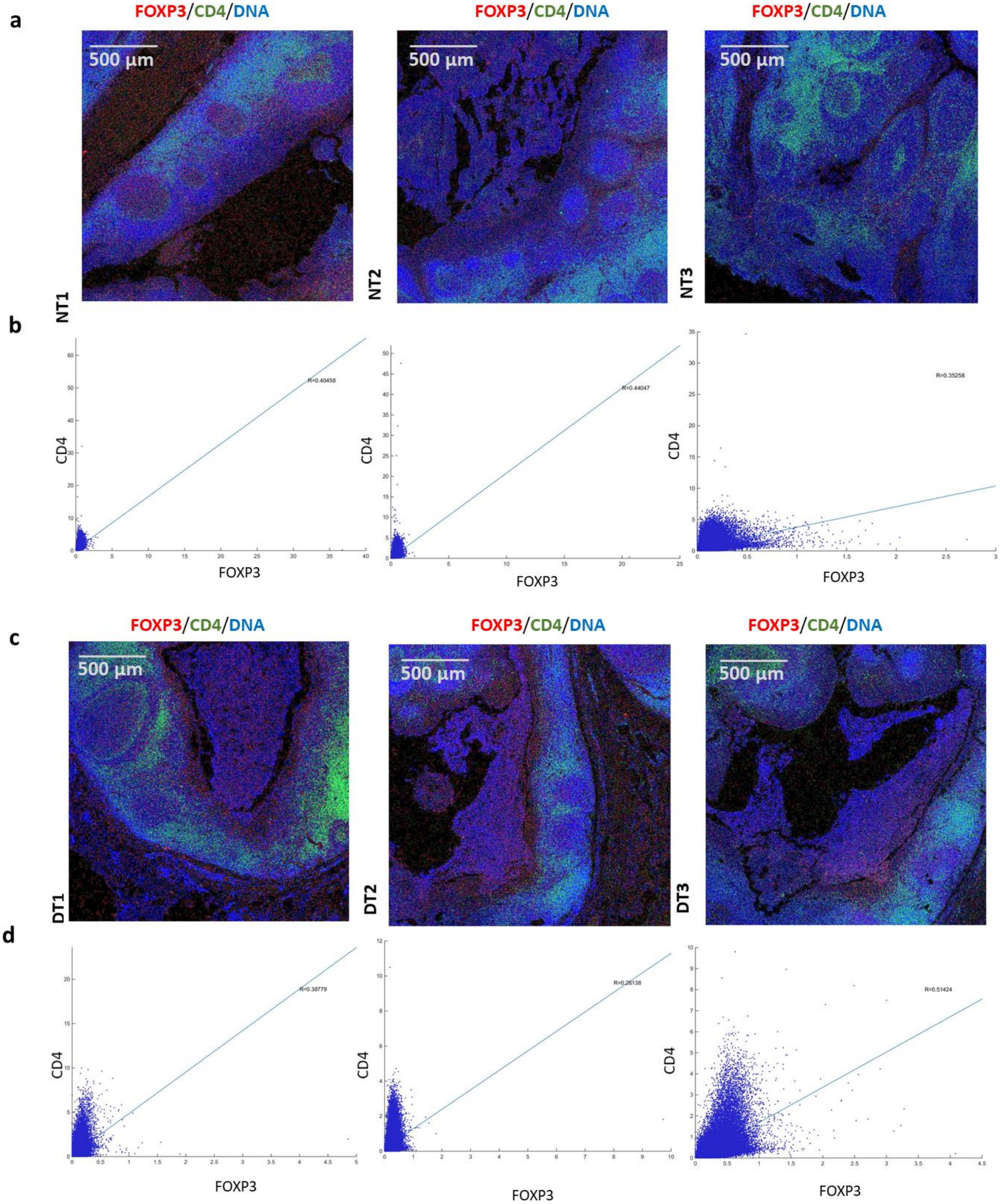
a
FOXP3/CD4/DNA
500 µm
NT1
NT2
NT3
b
R=0.40458
R=0.44047
R=0.35258
CD4
FOXP3
c
DT1
DT2
DT3
d
R=0.38779
R=0.26138
R=0.51424

Supplementary Figure 4. FOXP3+ cells Treg cells are a subset of CD4+ cells

a Visual representation demonstrated the co-expression of CD4 and FOXP3 in all three normal ROIs.

b Correlation analysis between CD4 and FOXP3 in all three normal ROIs was shown. Pearson's correlation coefficient was found to be R = 0.40458 for NT1, R = 0.44047 for NT2, and R = 0.35258 for NT3.

c Visual representation showed the co-expression of CD4 and FOXP3 in all three diseased ROIs

d Correlation analysis between CD4 and FOXP3 in all three diseased ROIs was presented. Pearson's correlation coefficient was found to be R = 0.38779 for DT1, R = 0.20138 for DT2, and R = 0.51424 for DT3.

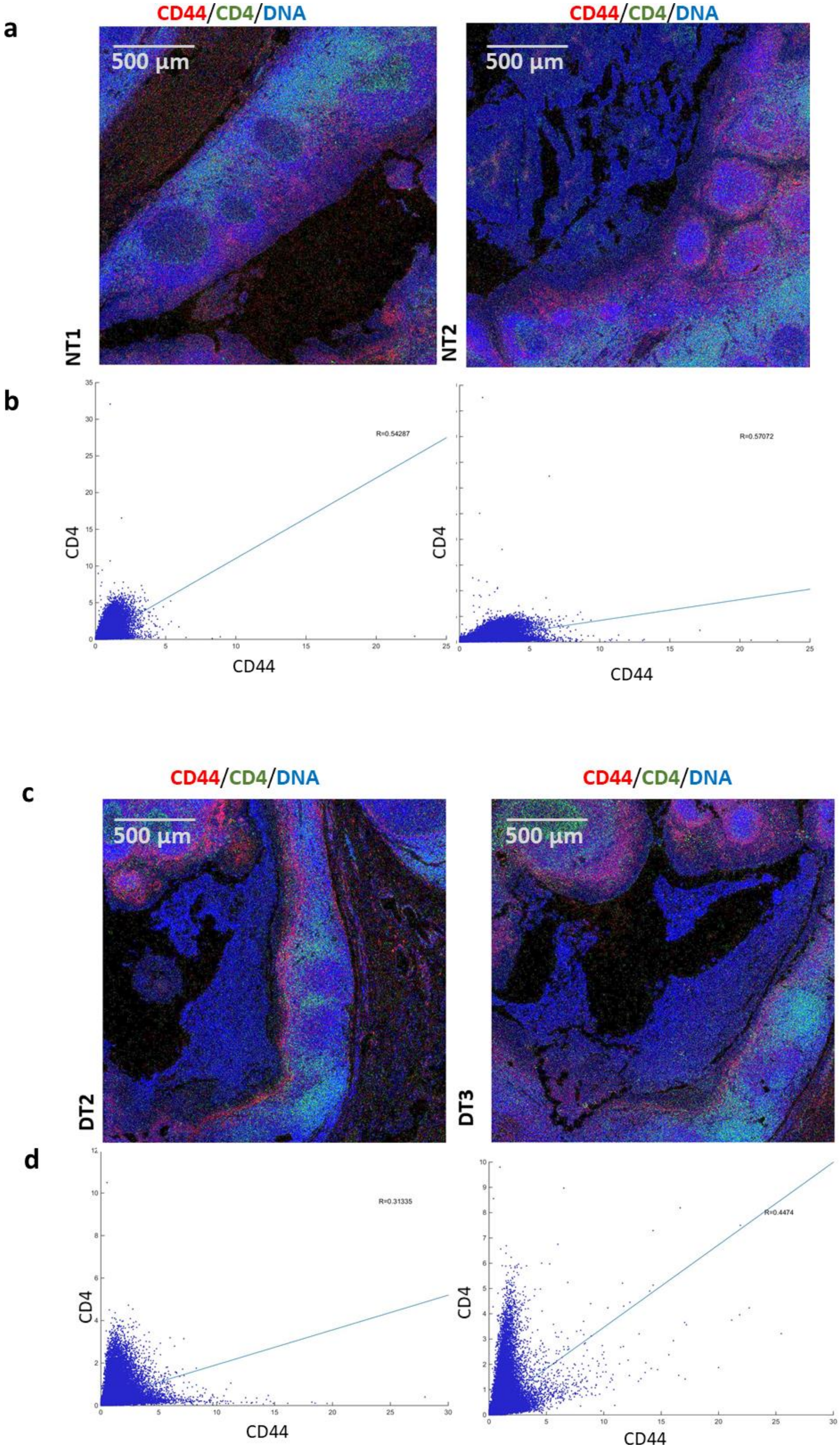
a
CD44/CD4/DNA
500 µm
NT1
CD44/CD4/DNA
500 µm
NT2
b
R=0.54287
CD4
CD44
R=0.57072
CD4
CD44
c
CD44/CD4/DNA
500 µm
DT2
CD44/CD4/DNA
500 µm
DT3
d
R=0.31335
CD4
CD44
R=0.4474
CD4
CD44

Supplementary Figure 5. CD44+ cells Treg cells are a subset of CD4+ cells

a Visual representation demonstrated the co-expression of CD4 and CD44 in all three normal ROIs.

b Correlation analysis between CD4 and CD44 in all three normal ROIs was presented. Pearson's correlation coefficient was found to be R = 0.54287 for NT1, and R = 0.57072 for NT2.

c Visual representation indicated the co-expression of CD4 and CD44 in all three diseased ROIs.

d Correlation analysis between CD4 and CD44 in all three diseased ROIs was shown. Pearson's correlation coefficient was found to be R = 0.31335 for DT2, and R = 0.4474 for DT3.

a

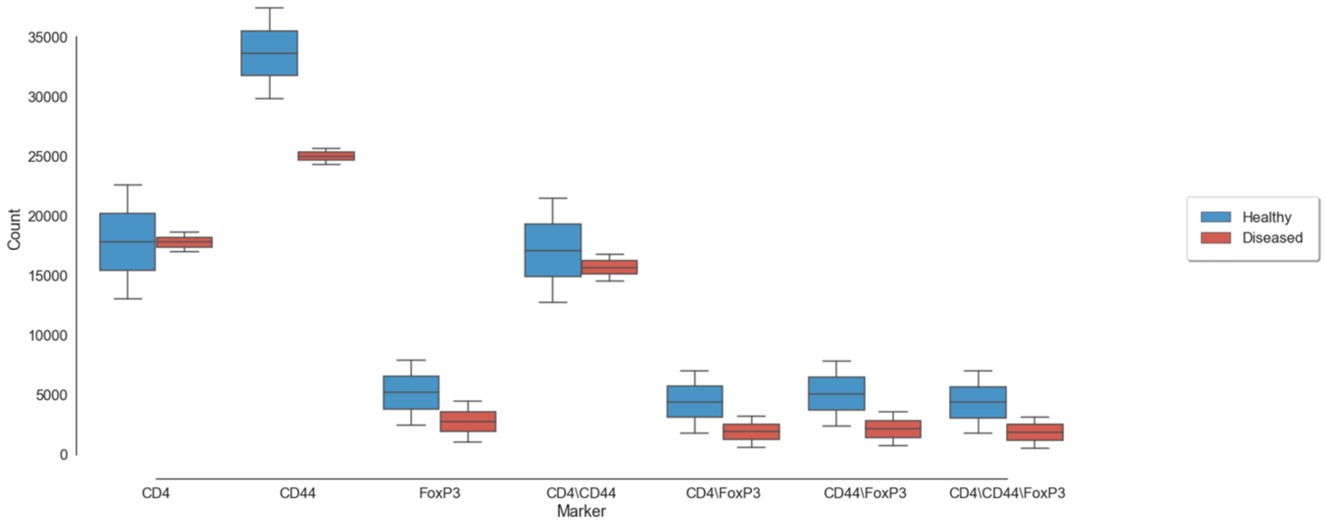
Count
35000
30000
25000
20000
15000
10000
5000
0
CD4
CD44
FoxP3
CD4\CD44
CD4\FoxP3
CD44\FoxP3
CD4\CD44\FoxP3
Marker
Healthy
Diseased

b

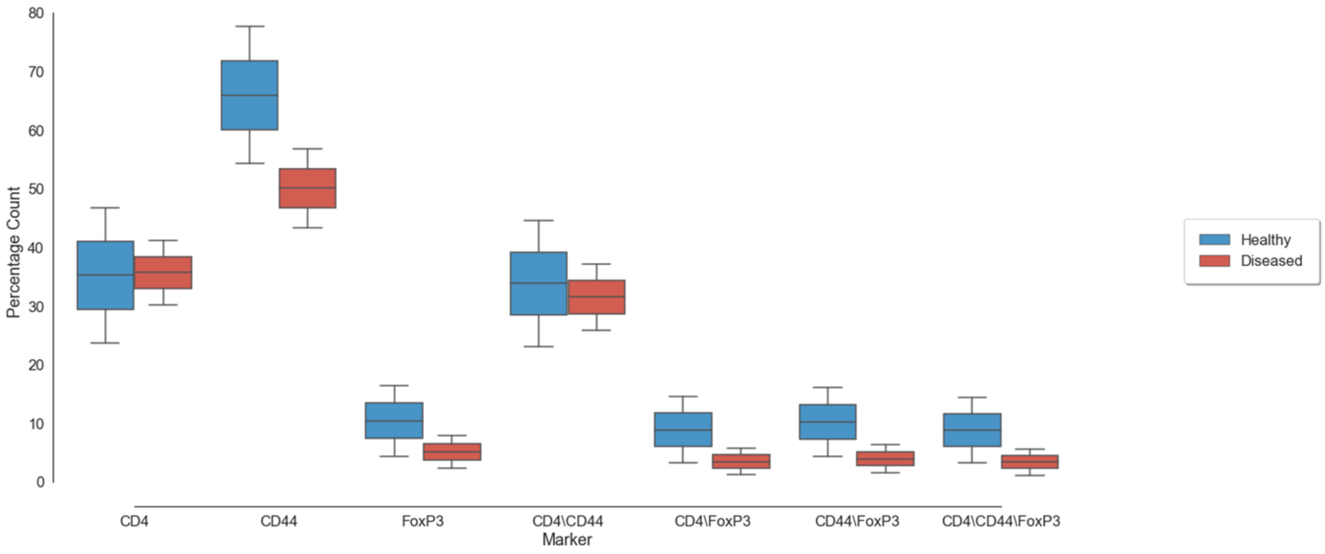
Percentage Count
80
70
60
50
40
30
20
10
0
CD4
CD44
FoxP3
CD4\CD44
CD4\FoxP3
CD44\FoxP3
CD4\CD44\FoxP3
Marker
Healthy
Diseased

Supplementary Figure 6. Treg cells were more active in the normal tonsil compared to diseased tonsil

a Box plot quantified the count of cells with positive staining with either CD4, FoxP3, CD44 alone, or different combinations to account for different regulatory T-cell phenotypes. Box plots show median first and third quartile, minimum and maximum (excluding outliers).

b Box plot indicated the percentage of cells in the ROIs with positive staining with either CD4, FoxP3, CD44 alone, or various combinations to account for different regulatory T-cell phenotypes. Box plots show median first and third quartile, minimum and maximum (excluding outliers).

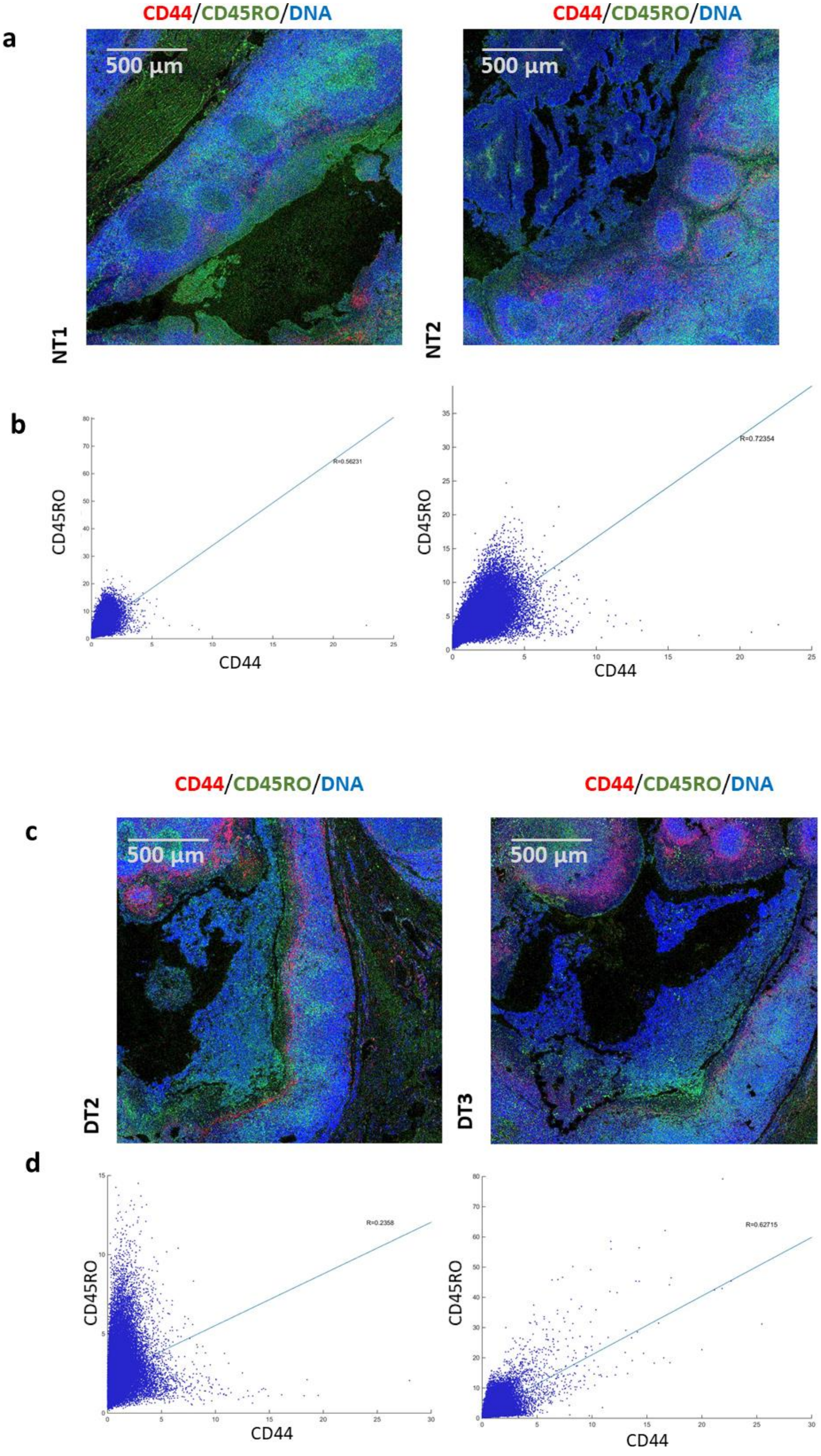
a
CD44/CD45RO/DNA
CD44/CD45RO/DNA
500 µm
500 µm
NT1
NT2
b
CD45RO
CD44
R=0.56231
CD45RO
CD44
R=0.72354
c
CD44/CD45RO/DNA
CD44/CD45RO/DNA
500 µm
500 µm
DT2
DT3
d
CD45RO
CD44
R=0.2358
CD45RO
CD44
R=0.62715

Supplementary Figure 7. Treg cells exhibited a memory phenotype.

a Visual representation showed the co-expression of CD44 and CD45RO across 3 ROIs of the normal tonsil.

b Correlation analysis between CD44 and CD45RO across 3 ROIs of the normal tonsil was presented. Pearson's correlation coefficient was found to be R = 0.56231 for NT1, and R = 0.72354 for NT2.

c Visual representation demonstrated the co-expression of CD44 and CD45RO across 3 ROIs of the diseased tonsil.

d Correlation analysis between CD44 and CD45RO across 3 ROIs of the diseased tonsil was shown. Pearson's correlation coefficient was found to be R = 0.2358 for DT2, and R = 0.62715 for DT3.

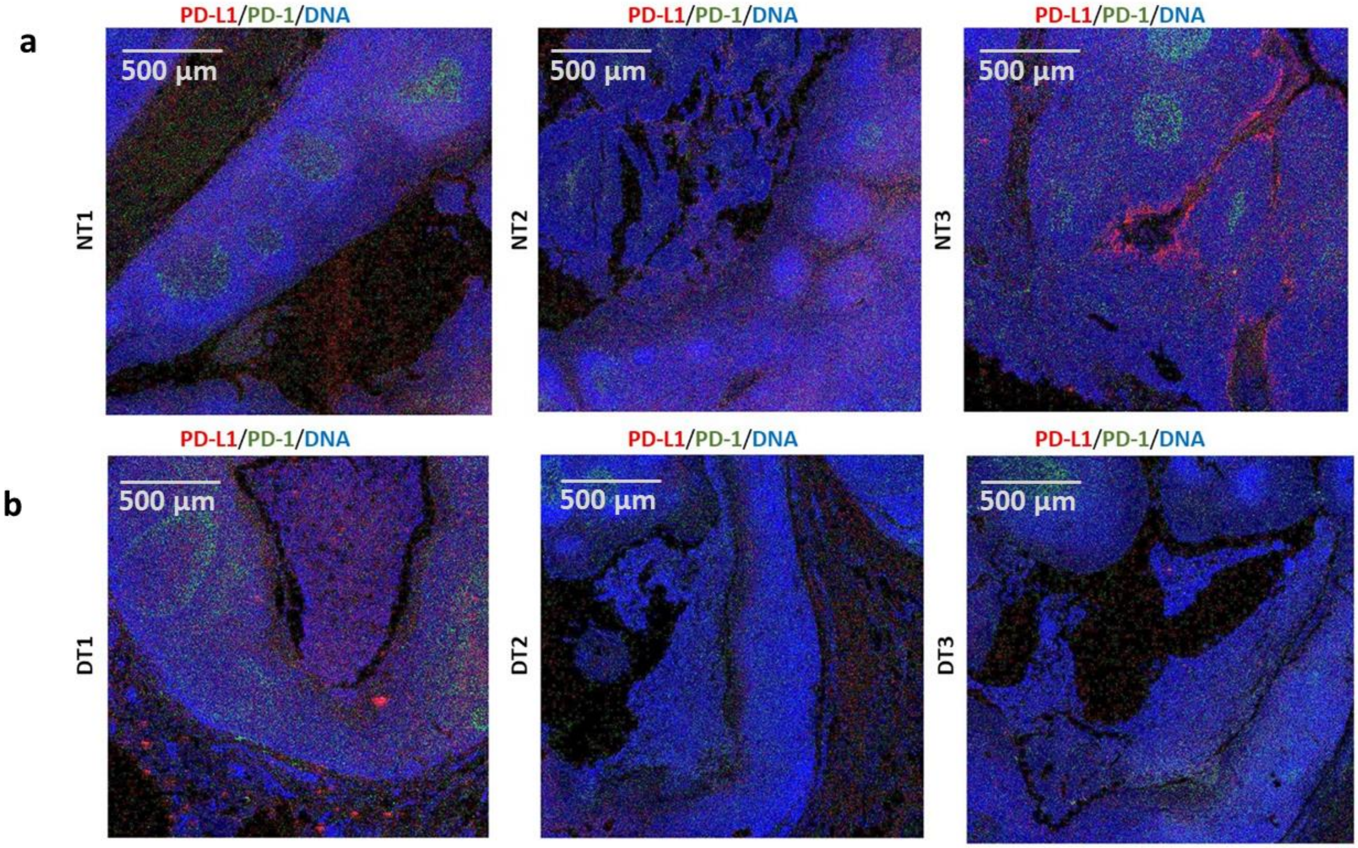
a
b
PD-L1/PD-1/DNA
500 µm
NT1
NT2
NT3
DT1
DT2
DT3

Supplementary Figure 8. PD-1 expressions were co-localized in the germinal centers

a Visual representation presented the co-expression of PD-1 and PD-L1 in all normal ROIs.

b Visual representation demonstrated the co-expression of PD-1 and PD-L1 in all diseased ROIs.

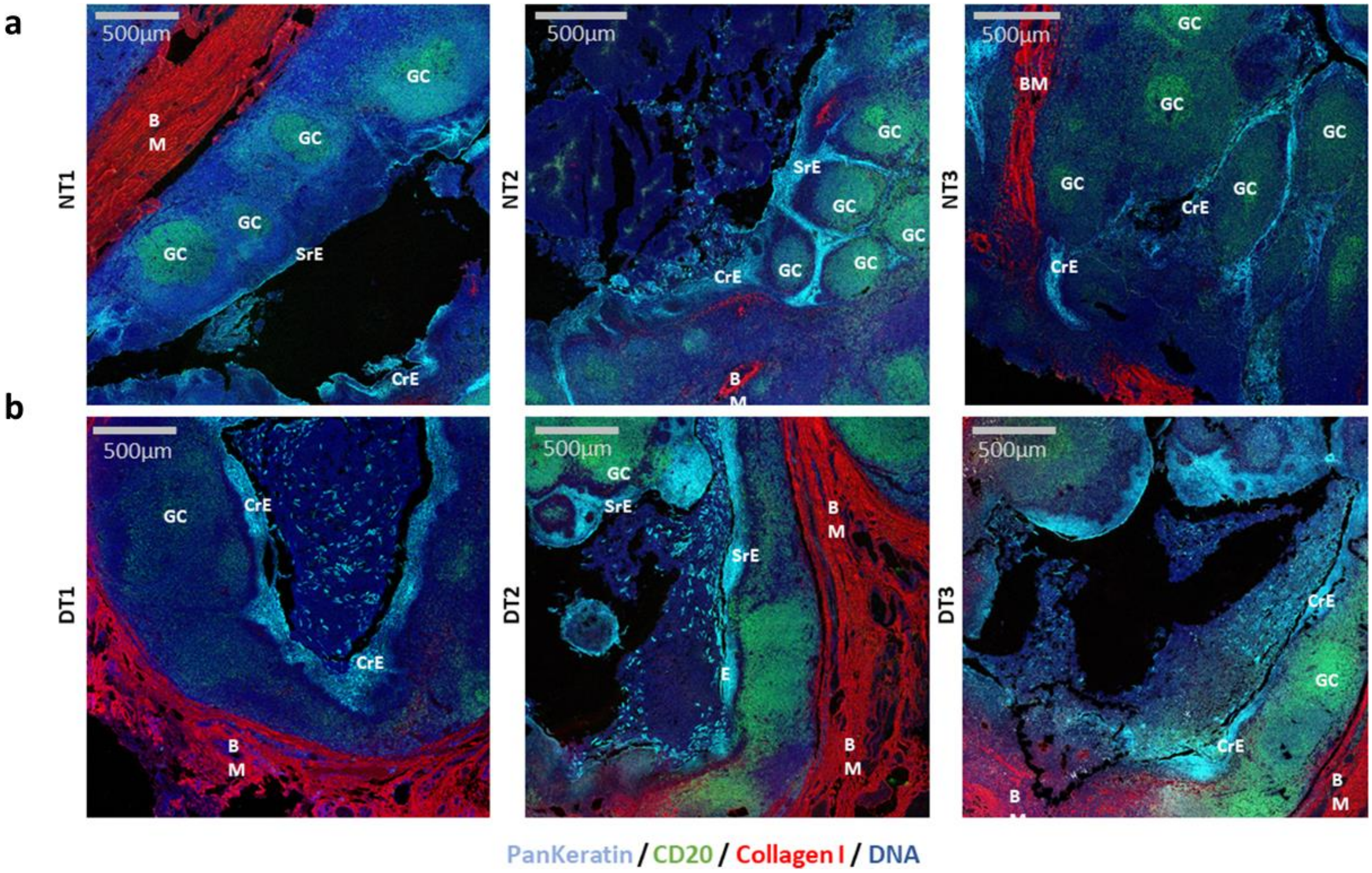
a
b
NT1
NT2
NT3
DT1
DT2
DT3
500µm
GC
B
M
BM
SrE
CrE
E
PanKeratin / CD20 / Collagen I / DNA

Supplementary Figure 9. Representative figures of normal and diseased tonsil tissues showed distinct anatomical regions.

a Representative composite of all regions of interest (ROIs) chosen for analysis in the normal and (b) diseased conditions was shown. BM (Basement membrane), GC (Germinal Center), and E (Epithelial cells) were denoted on each image. The continuous expression of collagen type 1 (shown in red) was used to identify the basement membrane, whereas PanKeratin (shown in cyan) was utilized to determine the epithelial cell layer. CD20 (shown in green) was used to identify the germinal center where B cells resided. The epithelial layer was further divided into SrE (stromal Epithelium) or (CrE) Crypt Epithelium, depending on the epithelial layer's continuity.

DT1

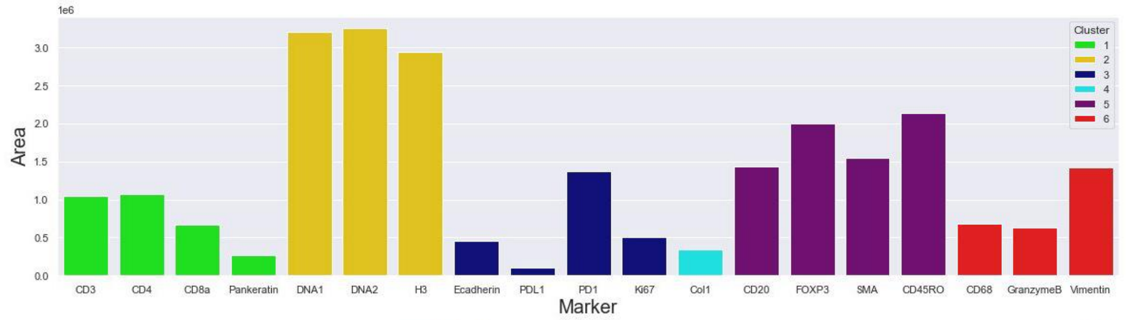

DT2

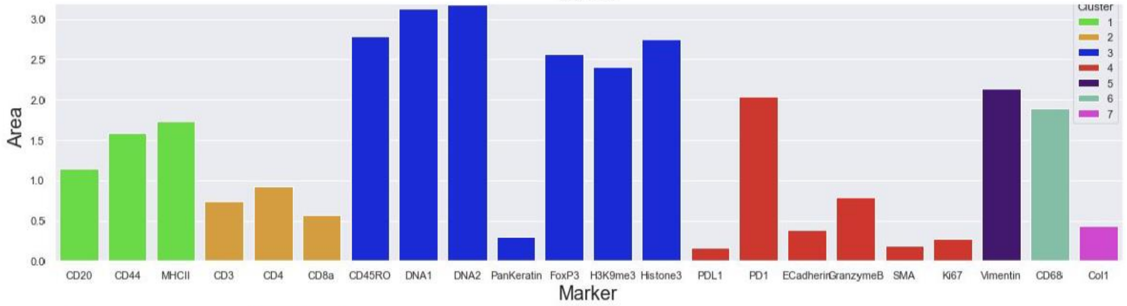

DT3

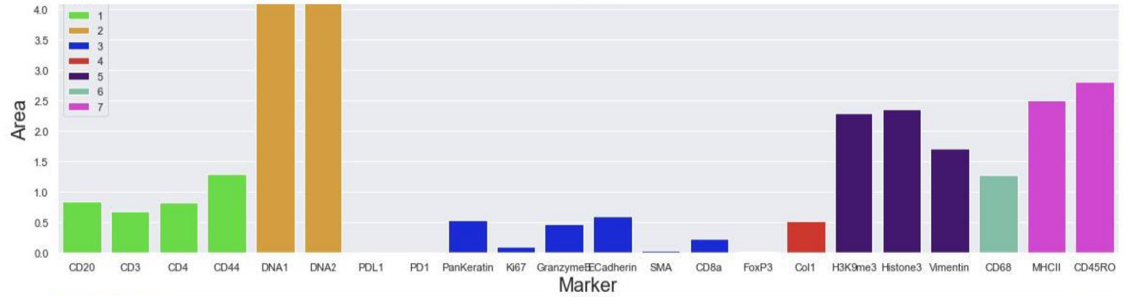

DT4

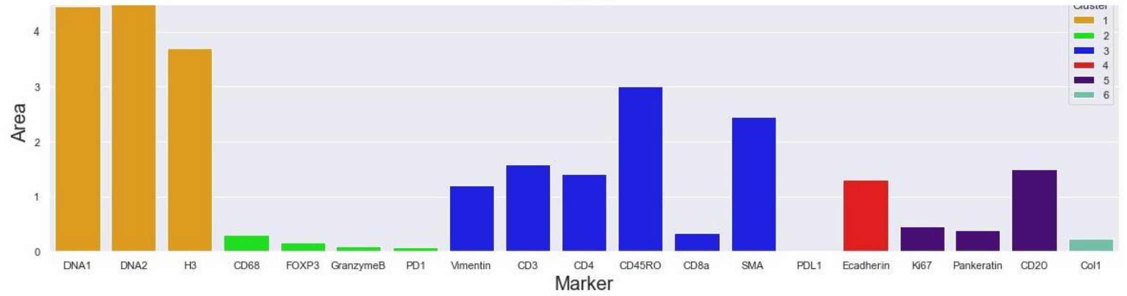

DT5

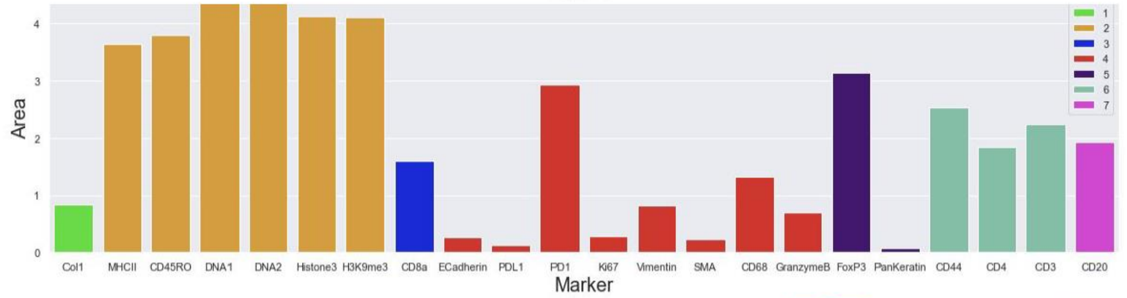

DT6

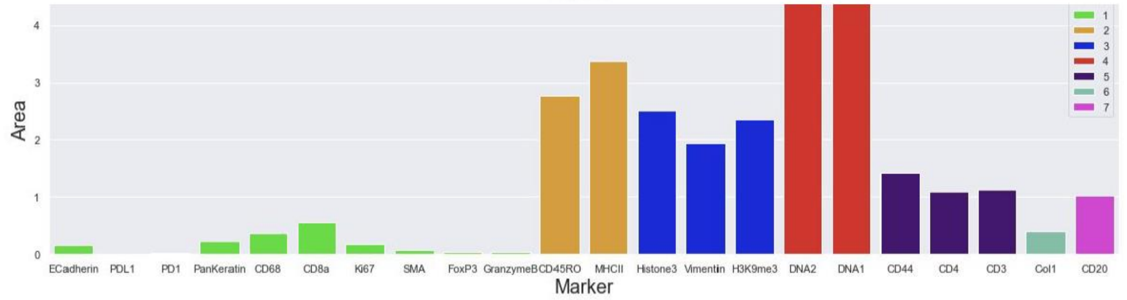

Supplementary Figure 10. Area ratio for markers in three tissues from the diseased tonsil.

Bar plots showed the area in the number of pixels of each marker in disease tonsil datasets. The color corresponding to the anatomical clustering results. DNA1, DNA2, and Histone 3 markers exhibited the highest area compared to other markers. Granzyme B's area coverage was higher in the first three datasets but relatively low compared to the last three.

NT1

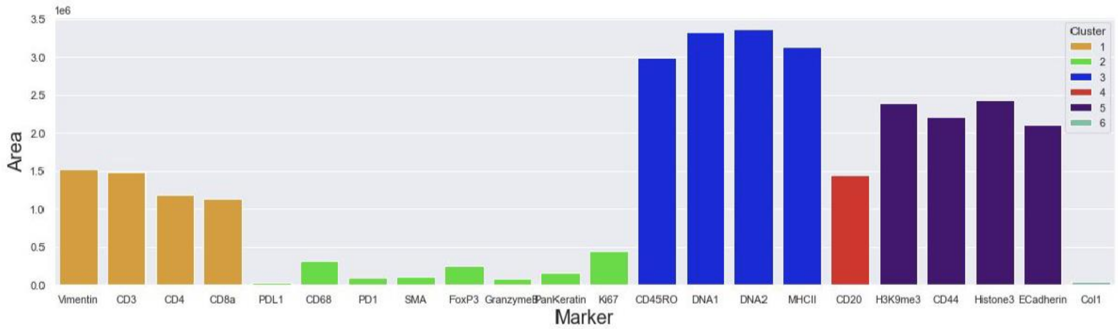

NT2

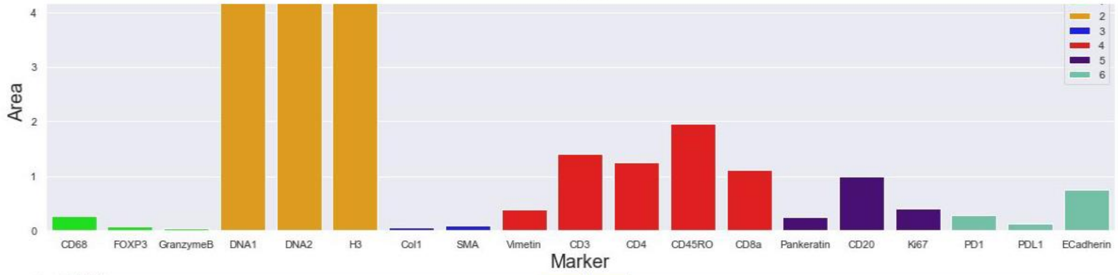

NT3

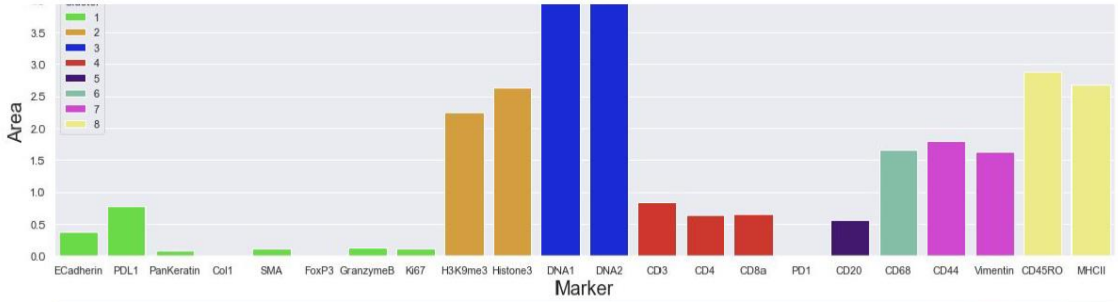

NT4

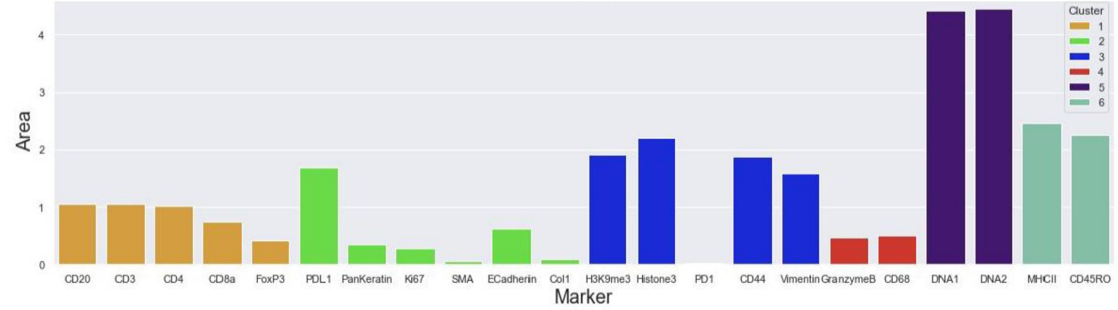

NT5

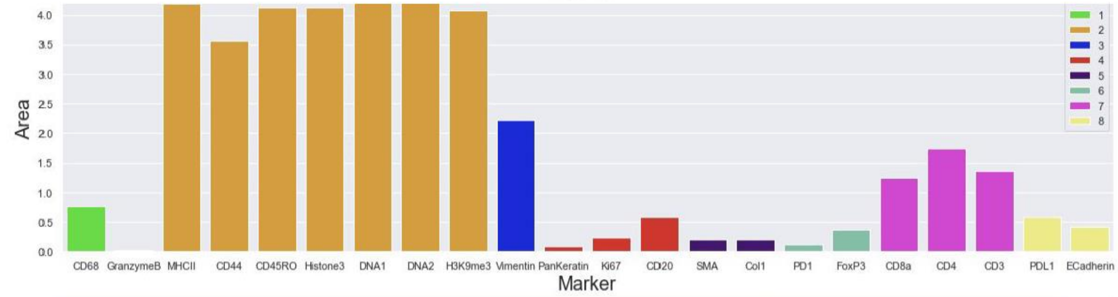

NT6

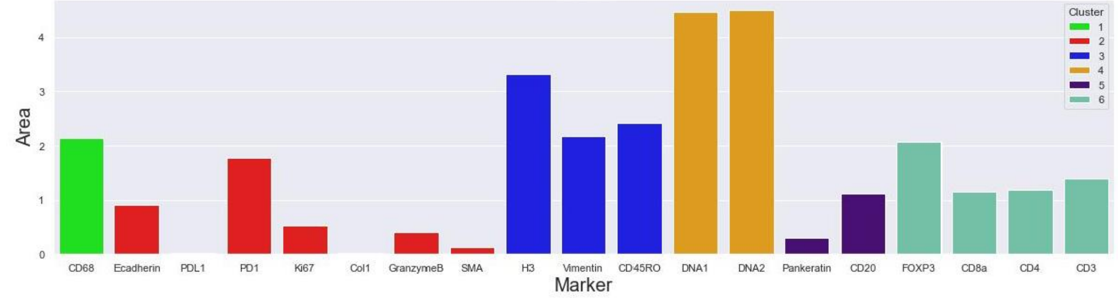

Supplementary Figure 11. Area ratio for markers in three tissues from the healthy tonsil.

Bar plots demonstrated the area in the number of pixels of each marker in normal tonsil datasets. The color corresponds to the anatomical clustering results. DNA1, DNA2, and Histone3 markers showed the highest area compared to other markers. The Granzyme B covered a small area in the images across all the datasets.

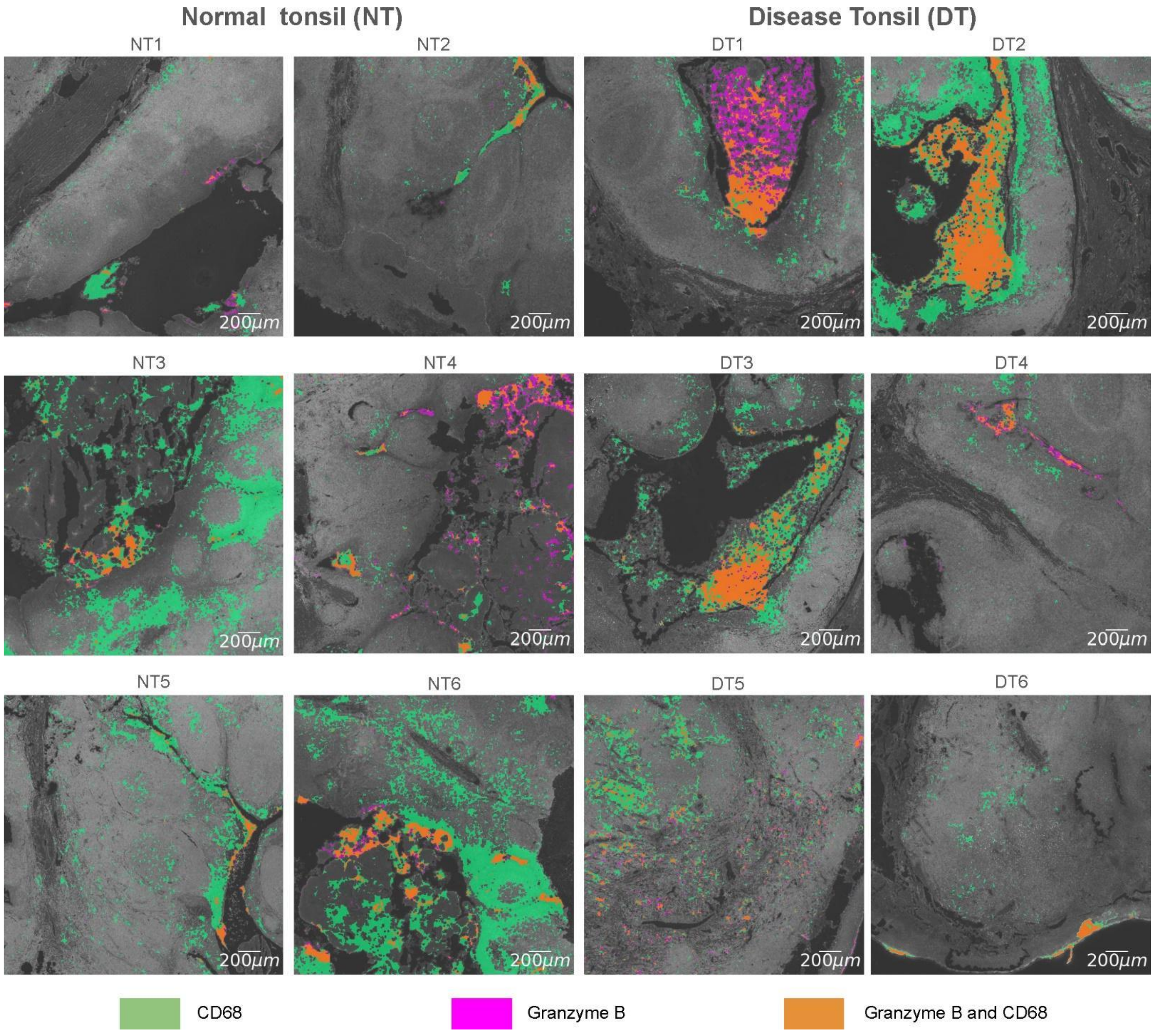

Normal tonsil (NT)
Disease Tonsil (DT)
NT1
NT2
DT1
DT2
NT3
NT4
DT3
DT4
NT5
NT6
DT5
DT6
200μm
CD68
Granzyme B
Granzyme B and CD68

Supplementary Figure 12. Granzyme B and CD68 marker co-expression region in disease and healthy datasets.

Area representation of Granzyme B and CD68 markers in all tonsil datasets was shown. Granzyme B positive areas in green and CD68 marker regions were represented in magenta. The co-expression of the two markers was denoted in orange. The scale bar is 200 μm

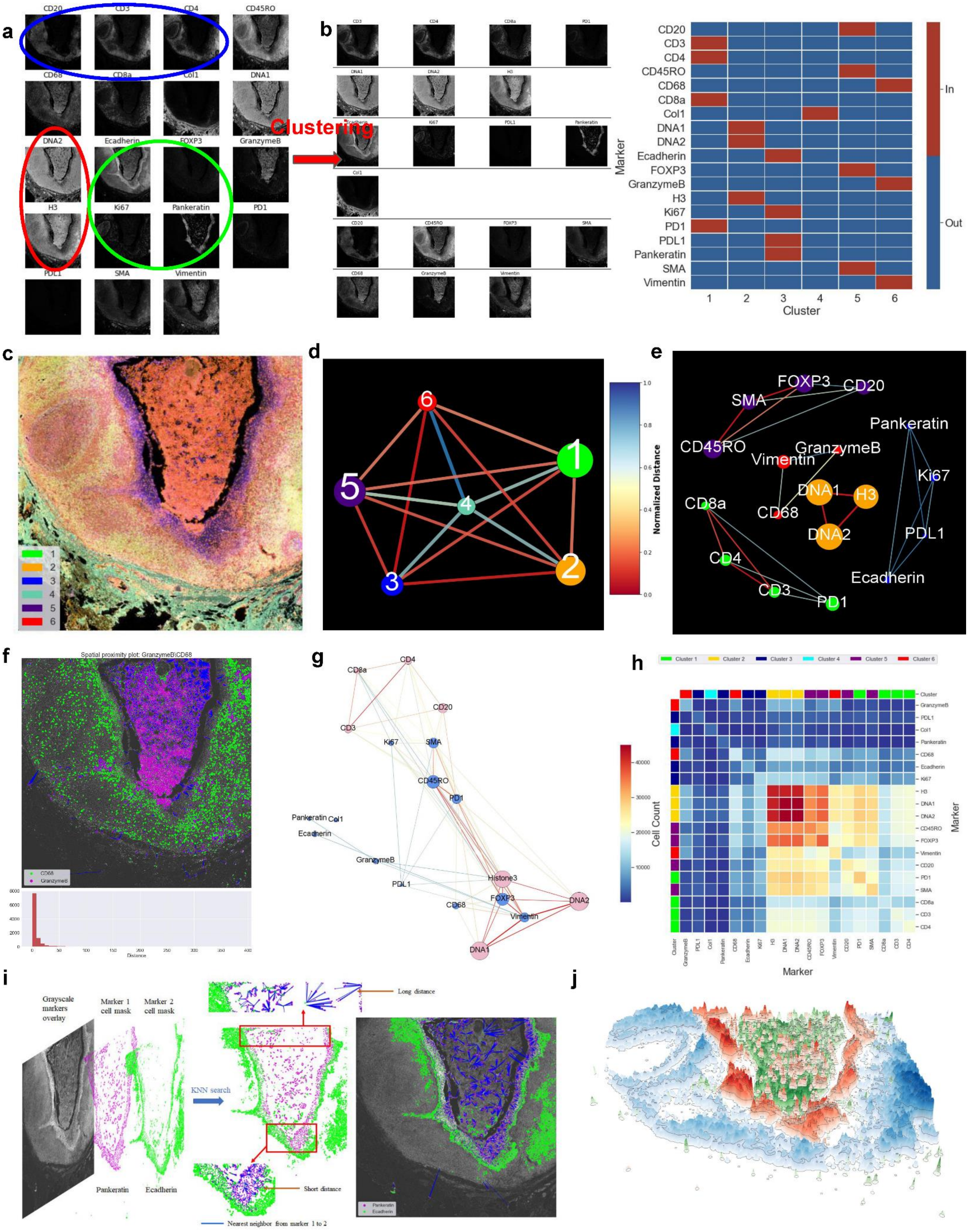
a
CD20
CD3
CD4
CD45RO
CD68
CD8a
Col1
DNA1
DNA2
Ecadherin
FOXP3
GranzymeB
H3
Ki67
Pankeratin
PD1
PDL1
SMA
Vimentin
Clustering
b
Marker
Cluster
1 2 3 4 5 6
In
Out
c
d
Normalized Distance
e
f
Spatial proximity plot: GranzymeB\CD68
CD68
GranzymeB
Distance
g
Histone3
h
Cluster 1
Cluster 2
Cluster 3
Cluster 4
Cluster 5
Cluster 6
Cell Count
Marker
i
Grayscale markers overlay
Marker 1 cell mask
Marker 2 cell mask
Pankeratin
Ecadherin
KNN search
Long distance
Short distance
Nearest neighbor from marker 1 to 2
j

Supplementary Figure 13. SpatialViz single-cell pathology of highly multiplexed protein maps in tonsils.

a Original multiplex images showed marker-specific expression level before clustering.

b Anatomical clustering from marker expression level was presented.

c Clustering anatomic visual by assigning a unique color to each cluster mean image was demonstrated.

d Inter-cluster distance network based on clustering result was displayed.

e Intra-cluster marker distance network based on clustering results was shown.

f Spatial proximity plot of Granzyme B and CD68 markers presented the co-expression and proximity.

g Landmark reference plot was displayed.

h Marker co-expression by cell count for all markers was shown.

i A schema showed the spatial proximity plot generation process.

j Topography plots of CD44, Granzyme B, and Pankeratin markers were displayed.

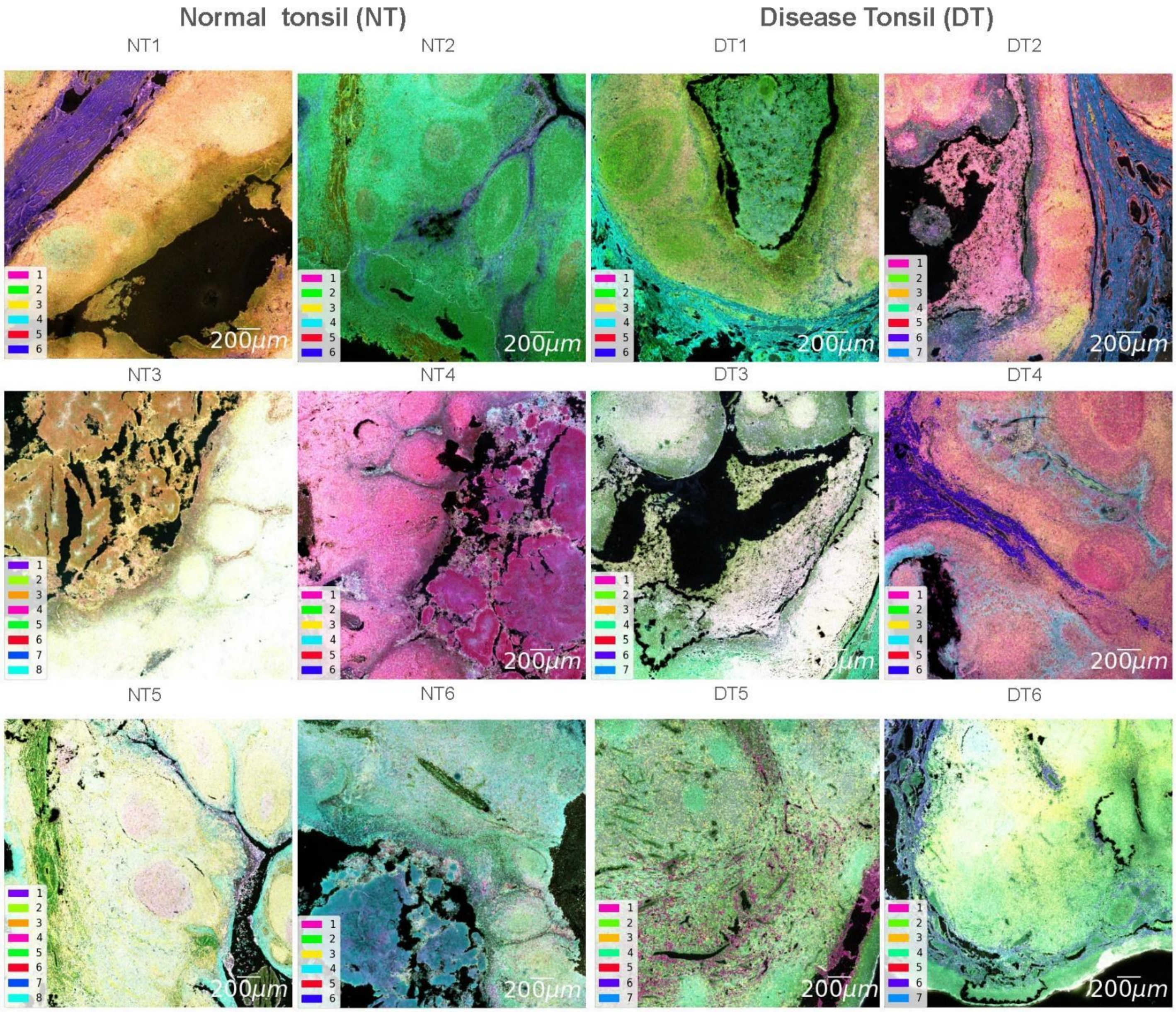
Normal tonsil (NT)
Disease Tonsil (DT)
NT1
NT2
DT1
DT2
NT3
NT4
DT3
DT4
NT5
NT6
DT5
DT6
200μm

Supplementary Figure 14. Multi-cluster representations of multiplex protein images in healthy and diseased tonsil tissues.

Cluster representations of all tonsil dataset images were shown.

Each tonsil dataset was clustered in an unsupervised manner.

The mean of each cluster image was colored and overlaid together.

The scale bar is 200 μm

DT1

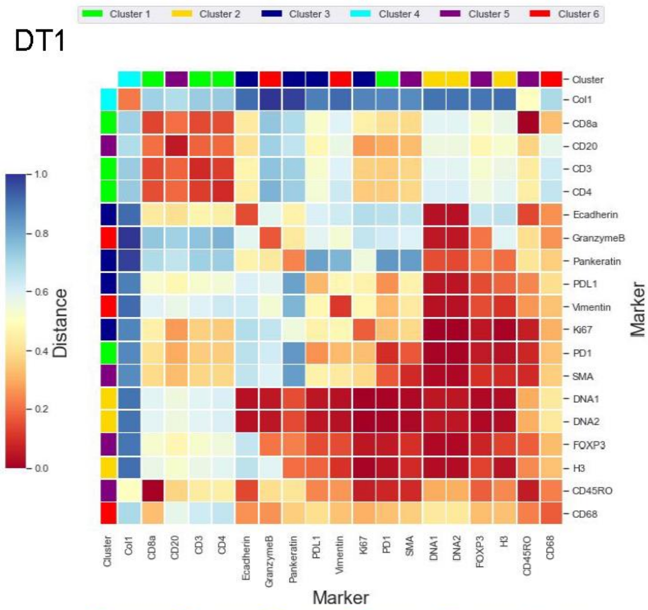

DT2

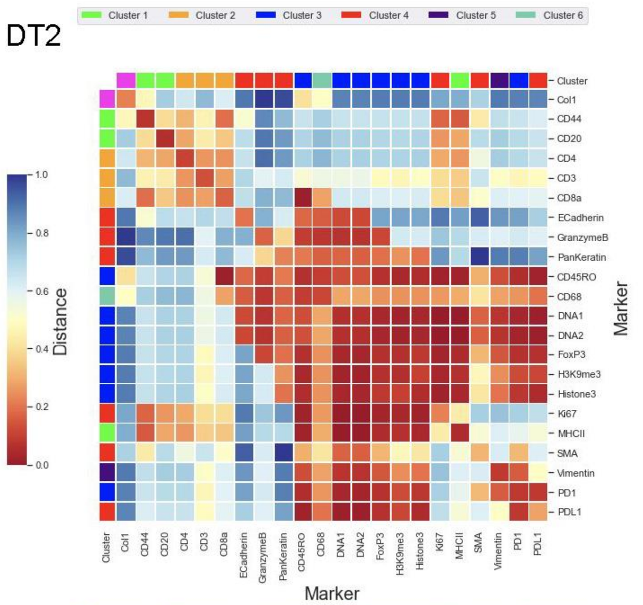

DT3

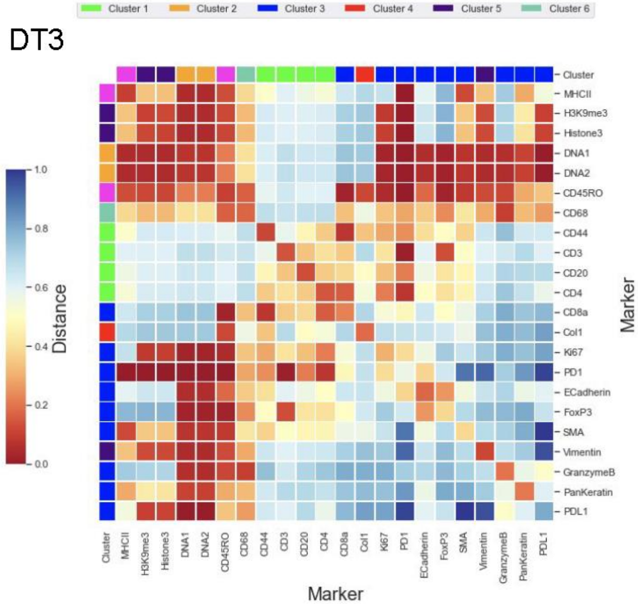

DT4

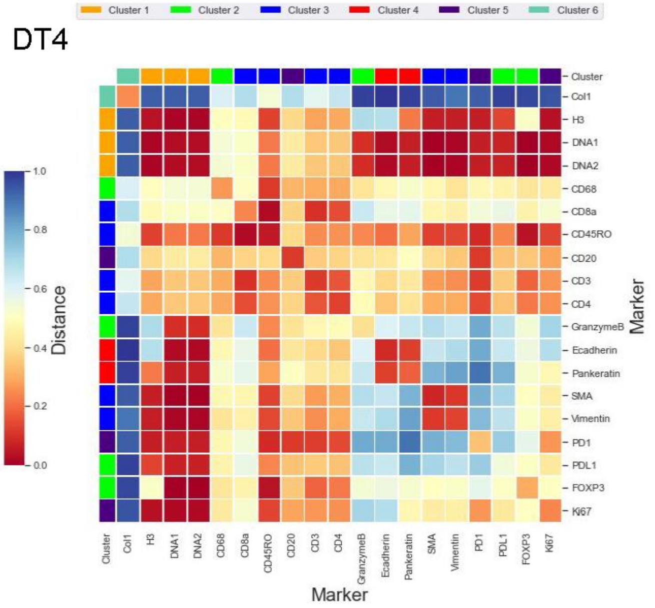

DT5

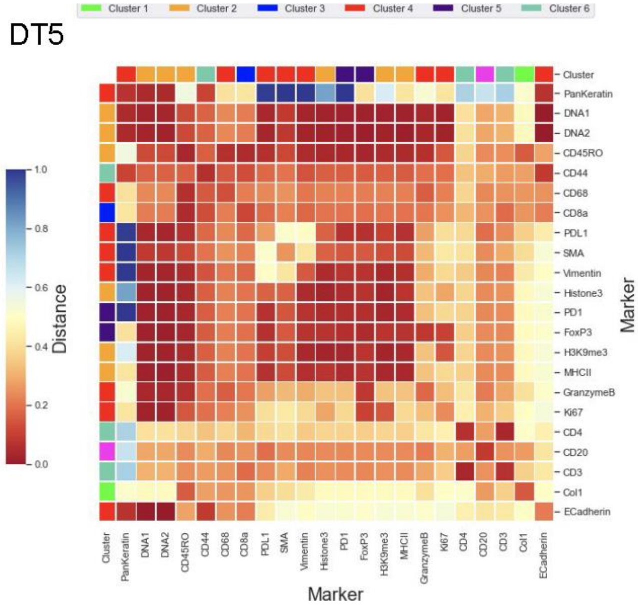

DT6

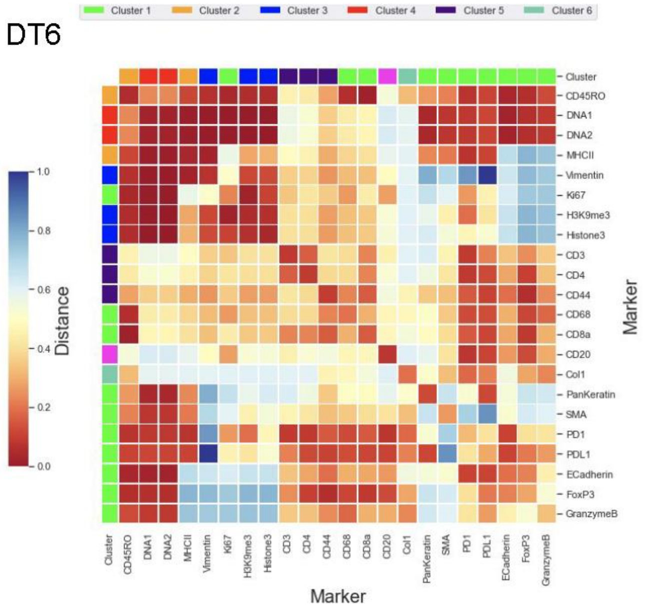

Supplementary Figure 15. Heatmaps of pairwise distances of markers in tissues from diseased tonsil.

Heatmaps represented the k-NN distance of cells between the pairs of markers in the diseased tonsil. The diagonal is the k-NN distance of the cells within a single marker that showed the marker expression's spread and density. The relative distances were normalized across the images. High overlapping regions such as DNA1, DNA2, and Histone 3 both exhibited short distances within the cells of the same marker and pair of markers.

NT1

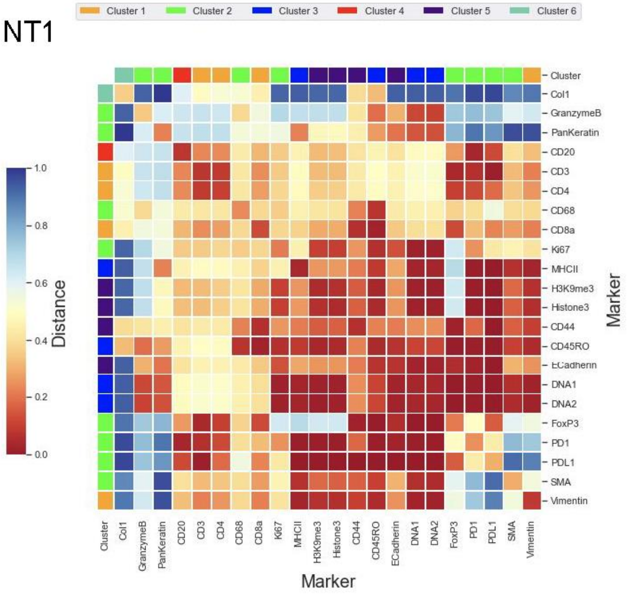

NT2

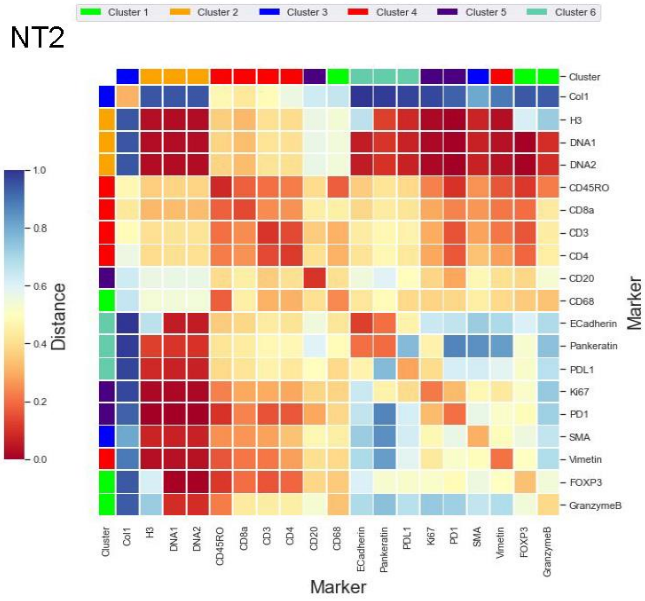

NT3

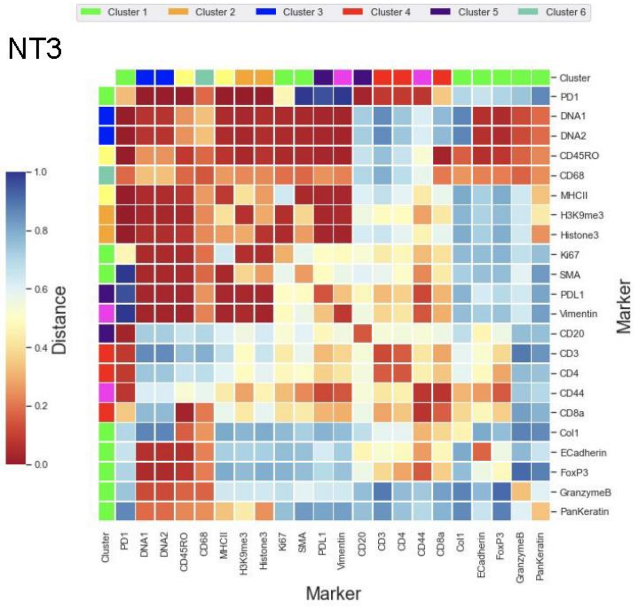

NT4

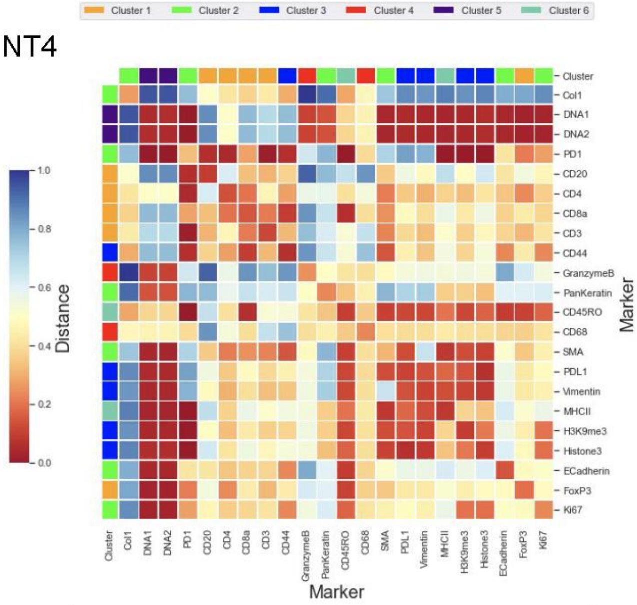

NT5

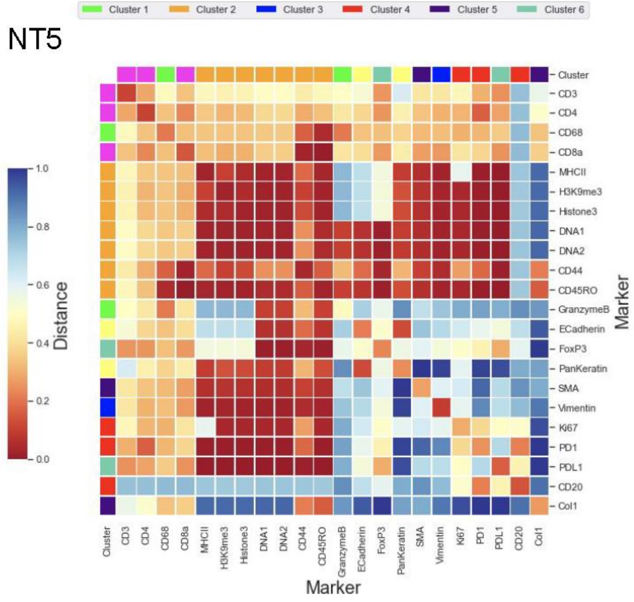

NT6

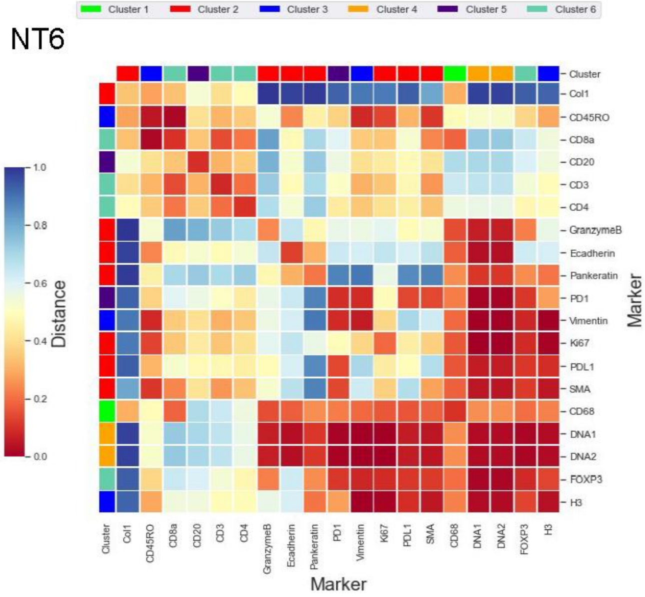

Supplementary Figure 16. Heatmaps of pairwise distances of markers in tissues from the healthy tonsil.

Heatmap showed the k-NN distances of cells between the pairs of markers in the healthy tonsil. The diagonal was the k-NN distance of cells within a single marker that demonstrated the marker expressions' spread and density. The relative distance was normalized across the images. High overlapping regions such as DNA 1, DNA 2, and Histone 3 both showed short distances within the cells of the same marker and pair of markers.

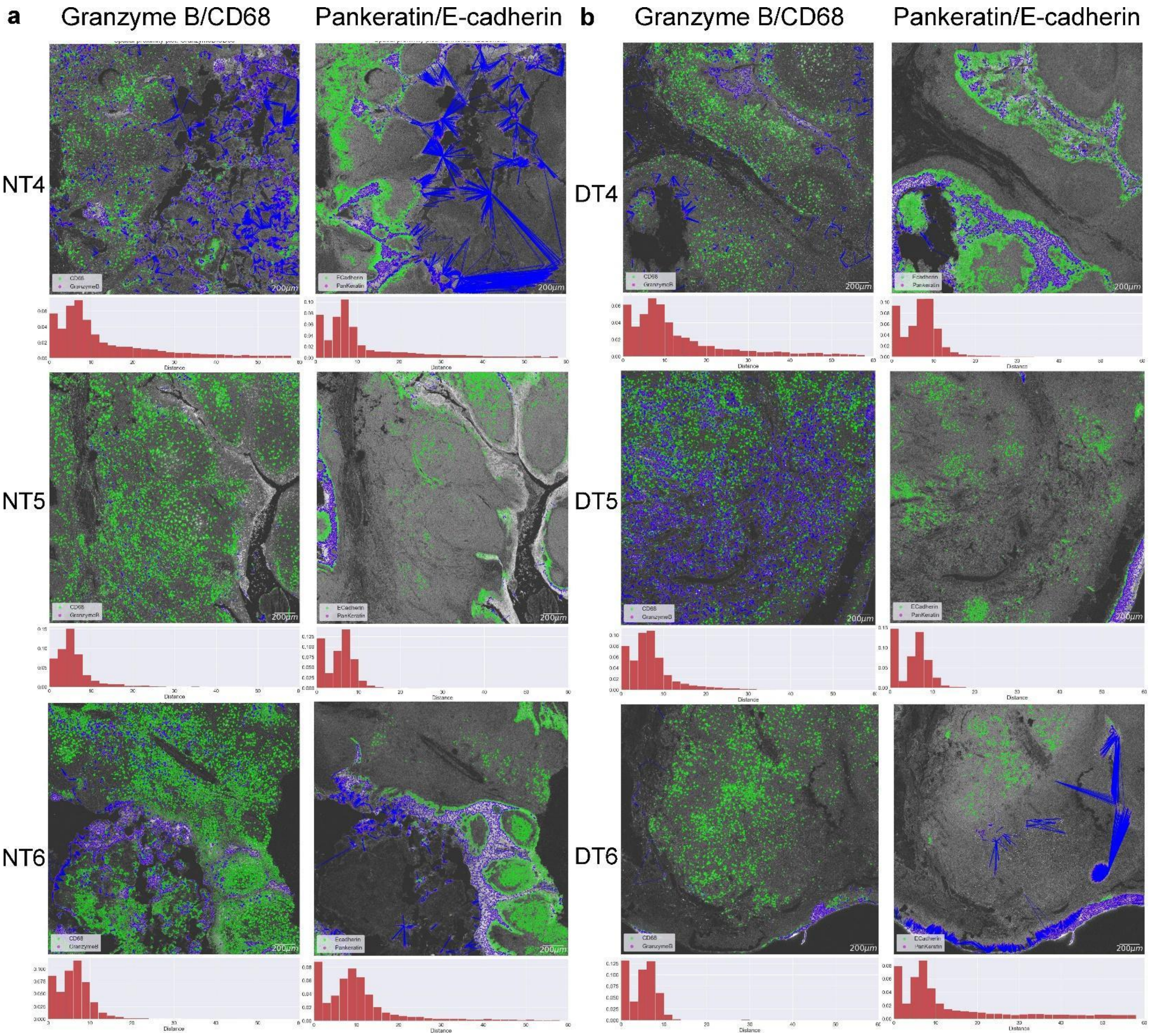
a
Granzyme B/CD68
Pankeratin/E-cadherin
b
Granzyme B/CD68
Pankeratin/E-cadherin
NT4
NT5
NT6
DT4
DT5
DT6
CD68
GranzymeB
ECadherin
PanKeratin
200μm
Distance

Supplementary Figure 17. Spatial proximity maps of cell-pairs within a k-neighbor distance (k-NN) in healthy and disease tonsils.

a Different spatial proximity map of cell pairs within nearest neighbor distance in healthy tonsil were shown. The scale bar is 200 μm

b Spatial proximity maps of cell pairs within the closest neighbor distance in healthy tonsils were demonstrated.

Magenta and green color represented individual cells in the pair of markers.

The presented plots corresponded to the Granzyme B/CD68 and Pankeratin/E-Cadherin pairs of the last three datasets of both diseased and healthy tonsils. The scale bar is 200 μm.

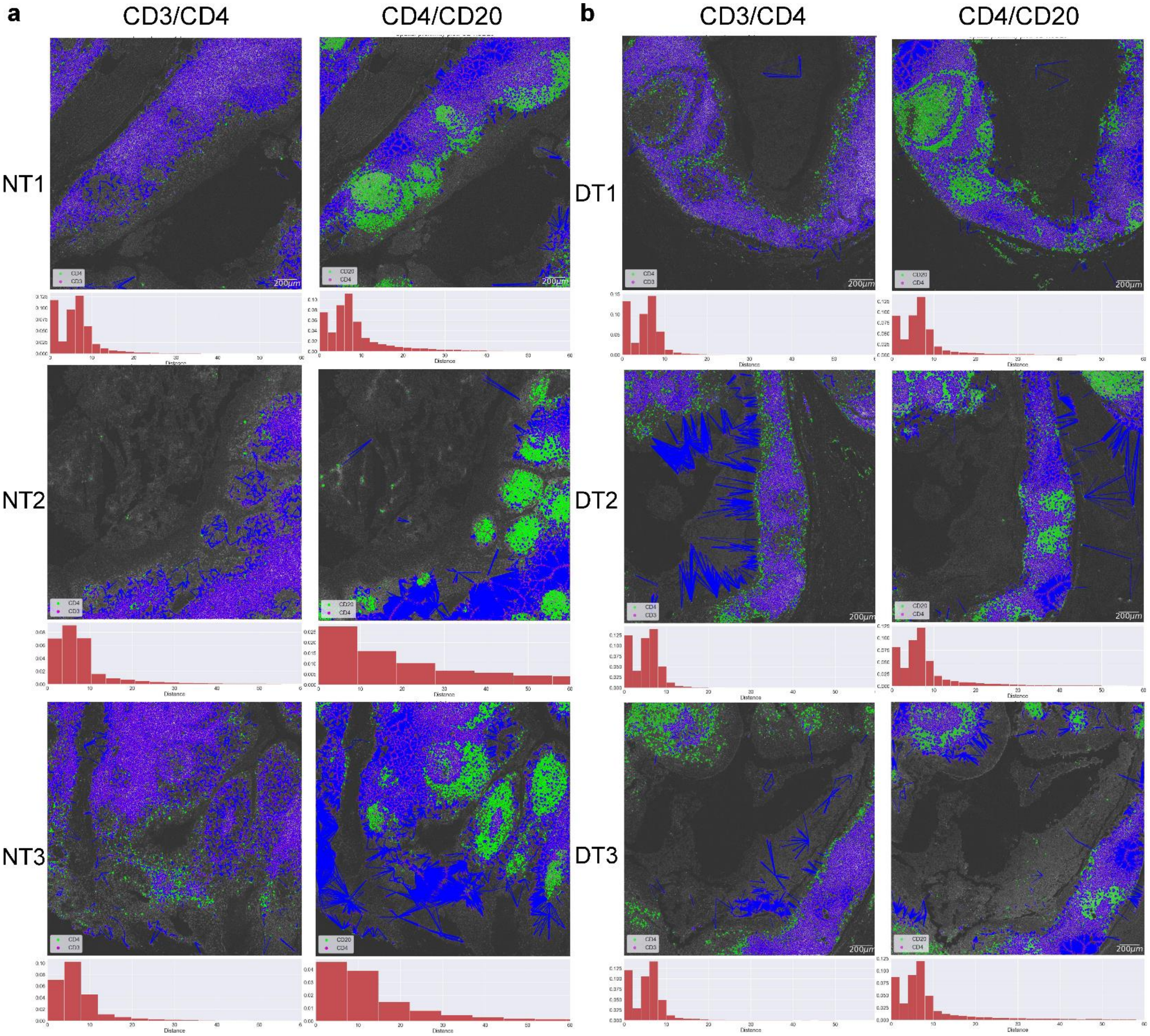
a
CD3/CD4
CD4/CD20
b
CD3/CD4
CD4/CD20
NT1
NT2
NT3
DT1
DT2
DT3
200μm
Distance

Supplementary Figure 18. Spatial proximity maps of cell pairs within a k-neighbor distance (k-NN) in healthy and disease tonsils for T cell marker pairs.

a Distinct spatial proximity maps of cell pairs within nearest neighbor distance in healthy tonsil were displayed. The scale bar is 200 μm

b Spatial proximity maps of cell pairs within closest neighbor distance in healthy tonsil were shown.

Magenta and green color denoted the individual cells in the pair of markers.

The plots showed the CD3/CD4 and CD4/CD20 pairs of the first three datasets of both diseased and healthy tonsils. The scale bar is 200 μm.

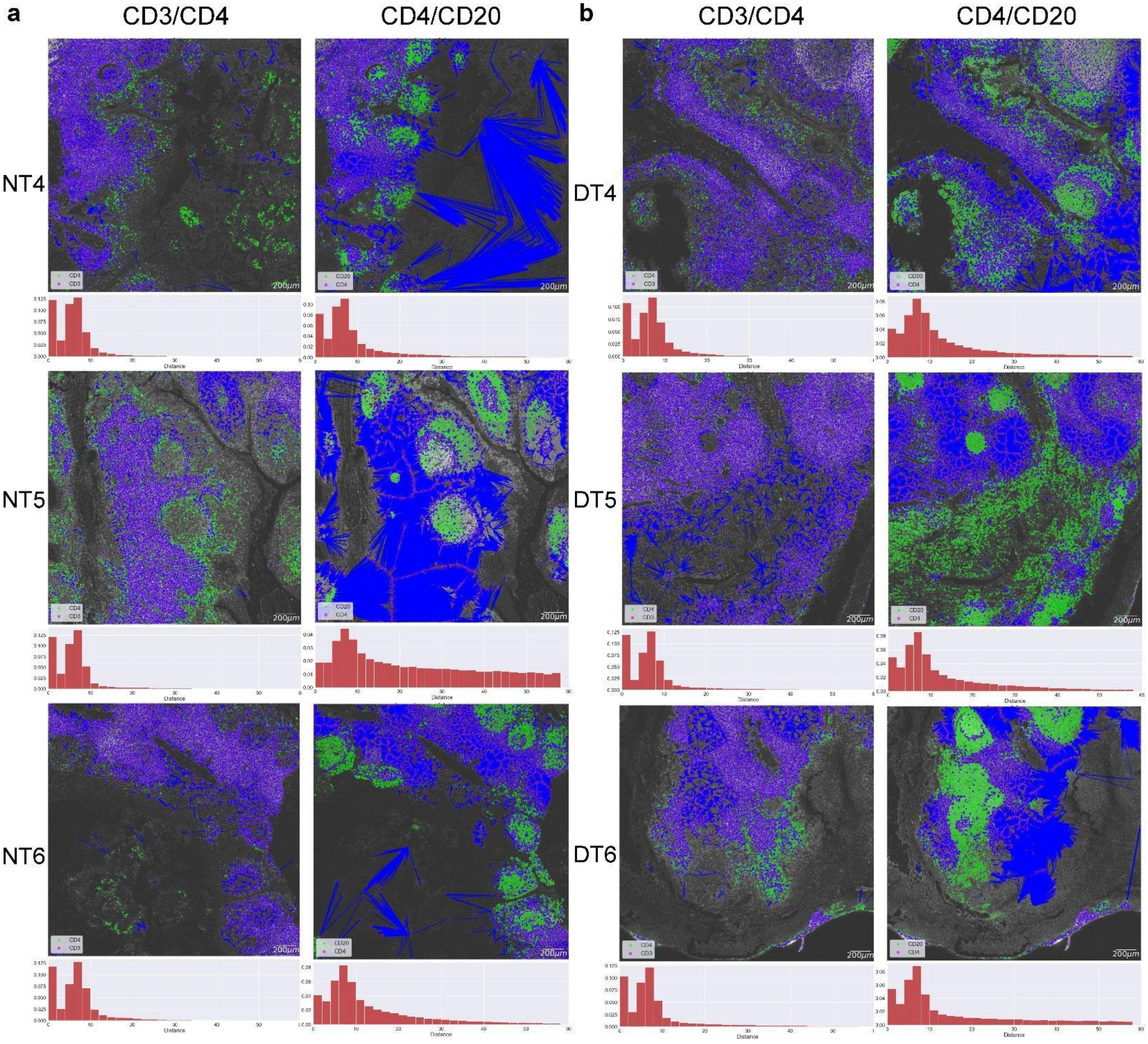
a
CD3/CD4
CD4/CD20
b
CD3/CD4
CD4/CD20
NT4
NT5
NT6
DT4
DT5
DT6
Distance

Supplementary Figure 19. Spatial proximity maps of cell pairs within a k-neighbor distance (k-NN) in healthy and disease tonsils for T cell marker pairs.

a A set of spatial proximity maps of cell pairs within nearest neighbor distance in healthy tonsil were shown.

b Spatial proximity maps of cell pairs within nearest neighbor distance in healthy tonsil were displayed.

Magenta and green color indicated the individual cells in the pair of markers.

The plots displayed the CD3/CD4 and CD4/CD20 pairs of the last three datasets of both diseased and healthy tonsils. The scale bar is 200 μm.

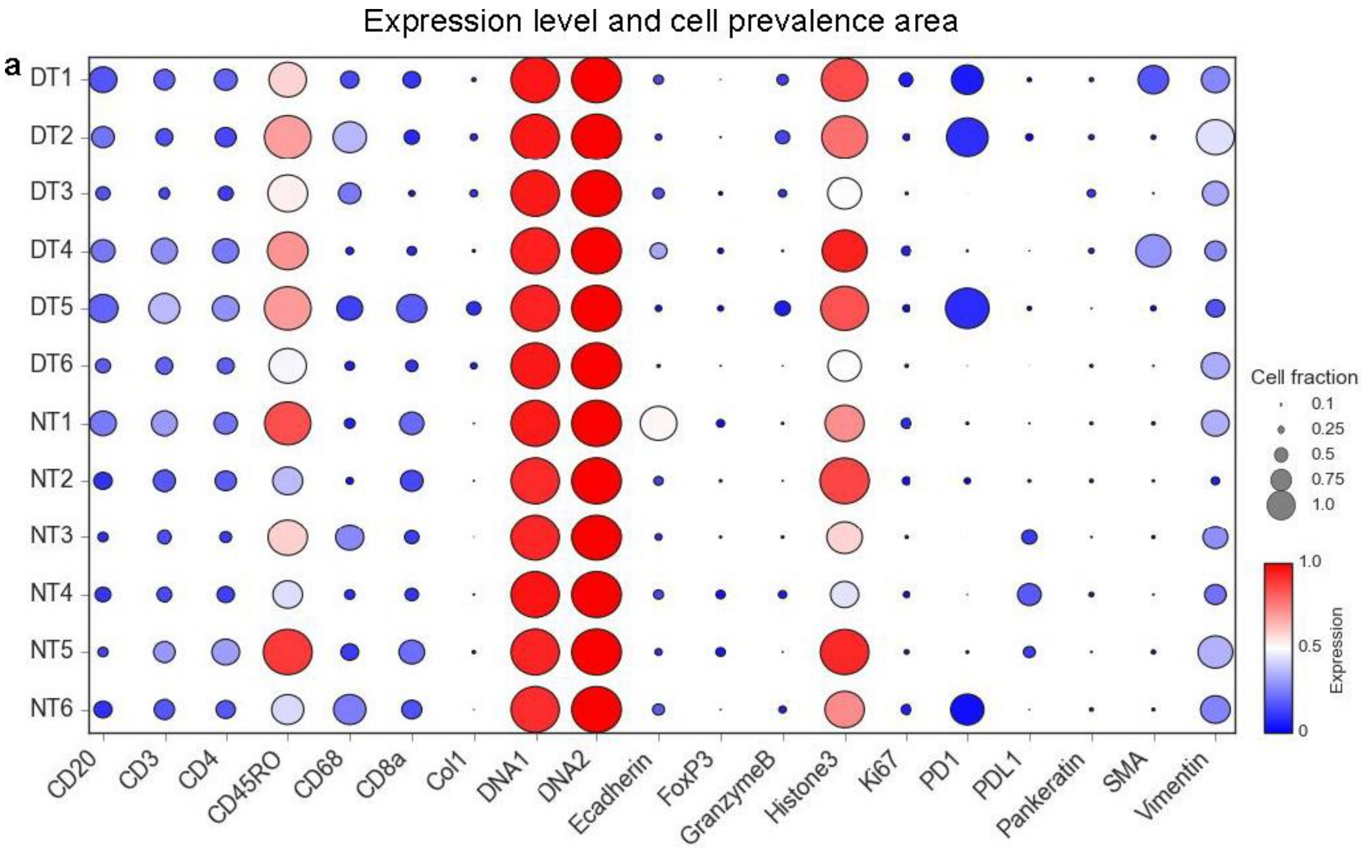
a
Expression level and cell prevalence area
DT1
DT2
DT3
DT4
DT5
DT6
NT1
NT2
NT3
NT4
NT5
NT6
CD20
CD3
CD4
CD45RO
CD68
CD8a
Col1
DNA1
DNA2
Ecadherin
FoxP3
GranzymeB
Histone3
Ki67
PD1
PDL1
Pankeratin
SMA
Vimentin
Cell fraction
0.1
0.25
0.5
0.75
1.0
Expression
1.0
0.5
0

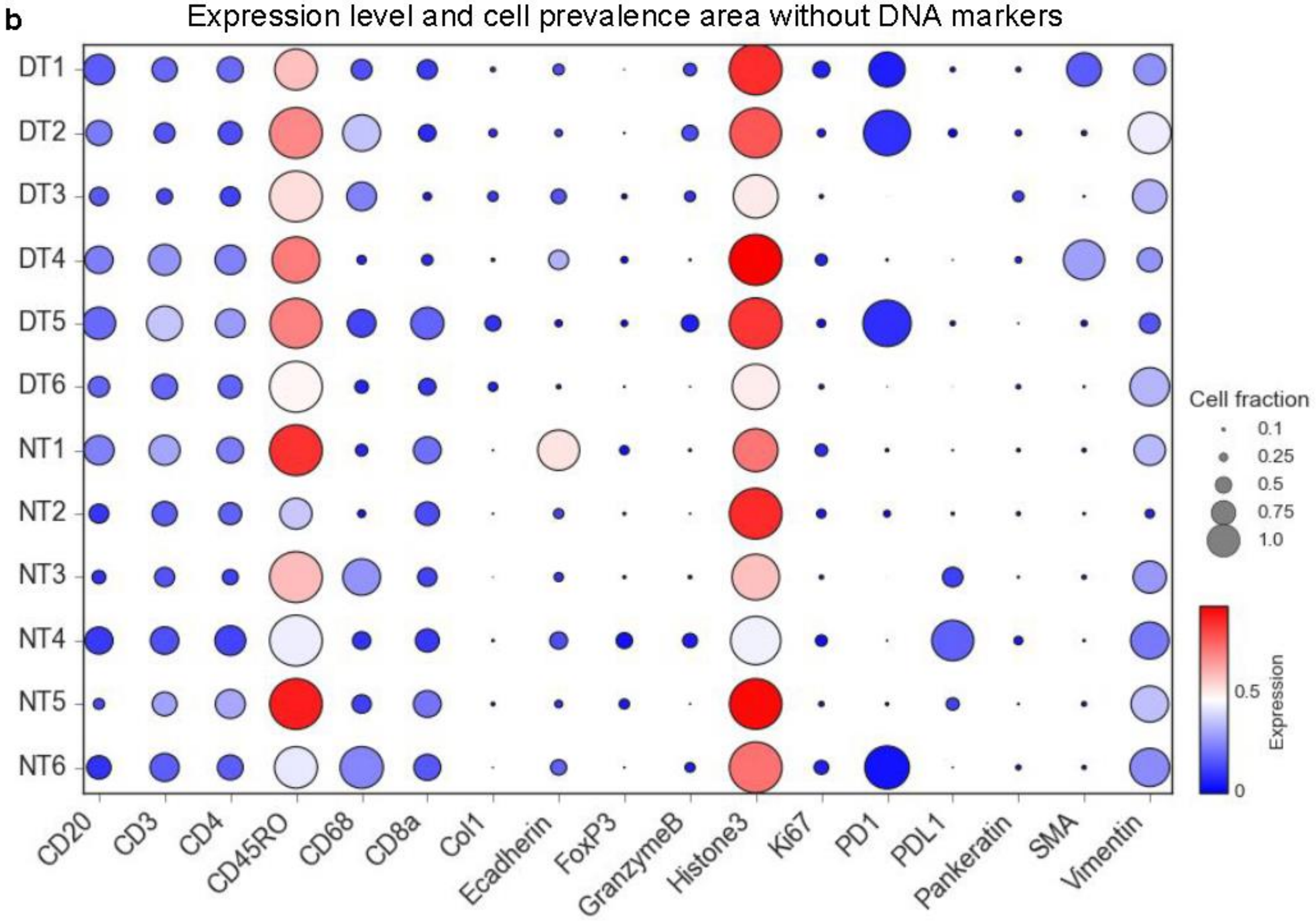
b
Expression level and cell prevalence area without DNA markers
DT1
DT2
DT3
DT4
DT5
DT6
NT1
NT2
NT3
NT4
NT5
NT6
CD20
CD3
CD4
CD45RO
CD68
CD8a
Col1
Ecadherin
FoxP3
GranzymeB
Histone3
Ki67
PD1
PDL1
Pankeratin
SMA
Vimentin
Cell fraction
0.1
0.25
0.5
0.75
1.0
Expression
0.5
0

Supplementary Figure 20. Dot plot representation of markers expression level and cell prevalence area

Dot plot representation of markers expression level and cell prevalence area for all 12 ROIs. The circle area represents the cell prevalence area of a specific marker, and the colormap represents the normalized expression level.

a Dot plot with DNA1 and DNA2 marker

b Dot plot without DNA1 and DNA2 marker

a Clustering expression level and cell prevalence area

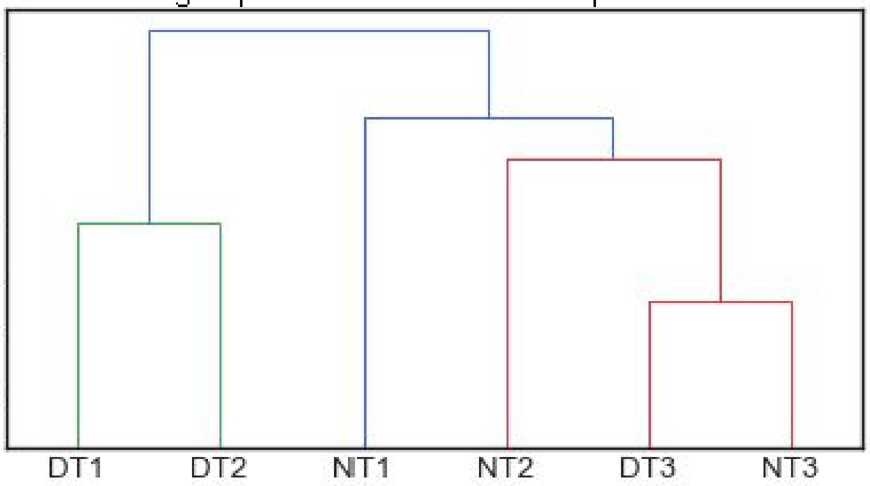

b Clustering expression level and cell prevalence area with only CD68, GranzymeB, Ki67, PD1, Pankeratin, SMA, Vimentin markers

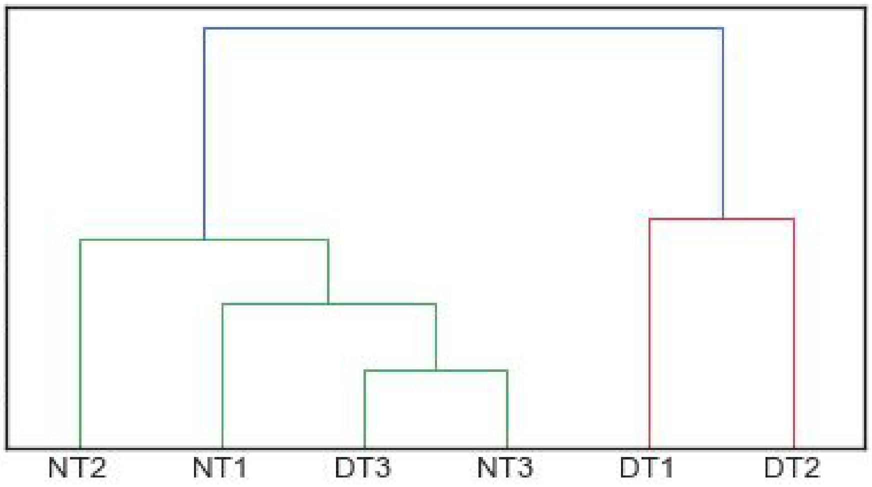

c Clustering pairwise marker mean cell-to-cell distance and fraction of distance inferior to 30 µm.

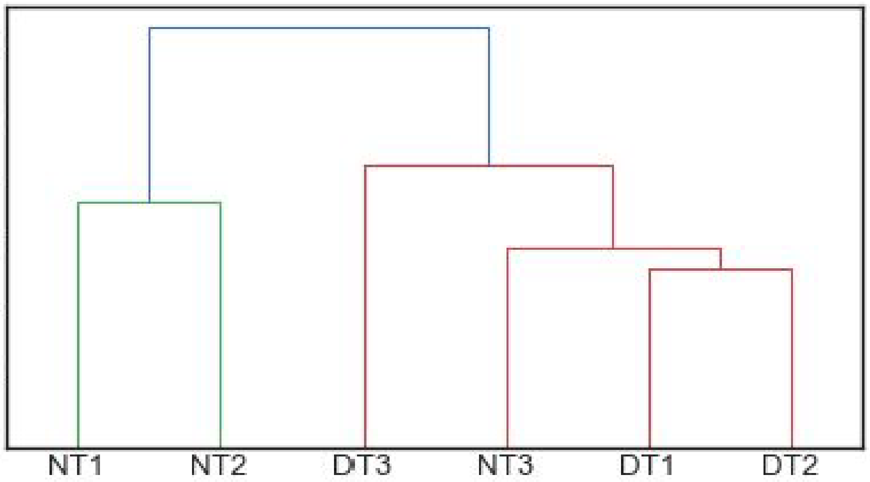

Supplementary Figure 21. Dataset clustering based on extracted features

Clustering was performed in three healthy and three disease datasets based on the following conditions.

a Markers expression level and cell prevalence area for all data were analyzed.

b Markers expression level and cell prevalence area using only CD68, GranzymeB, Ki67, PD1, Pankeratin, SMA, Vimentin markers were processed.

c Pairwise marker mean cell-to-cell distance and fraction of distance inferior to 30 μm.

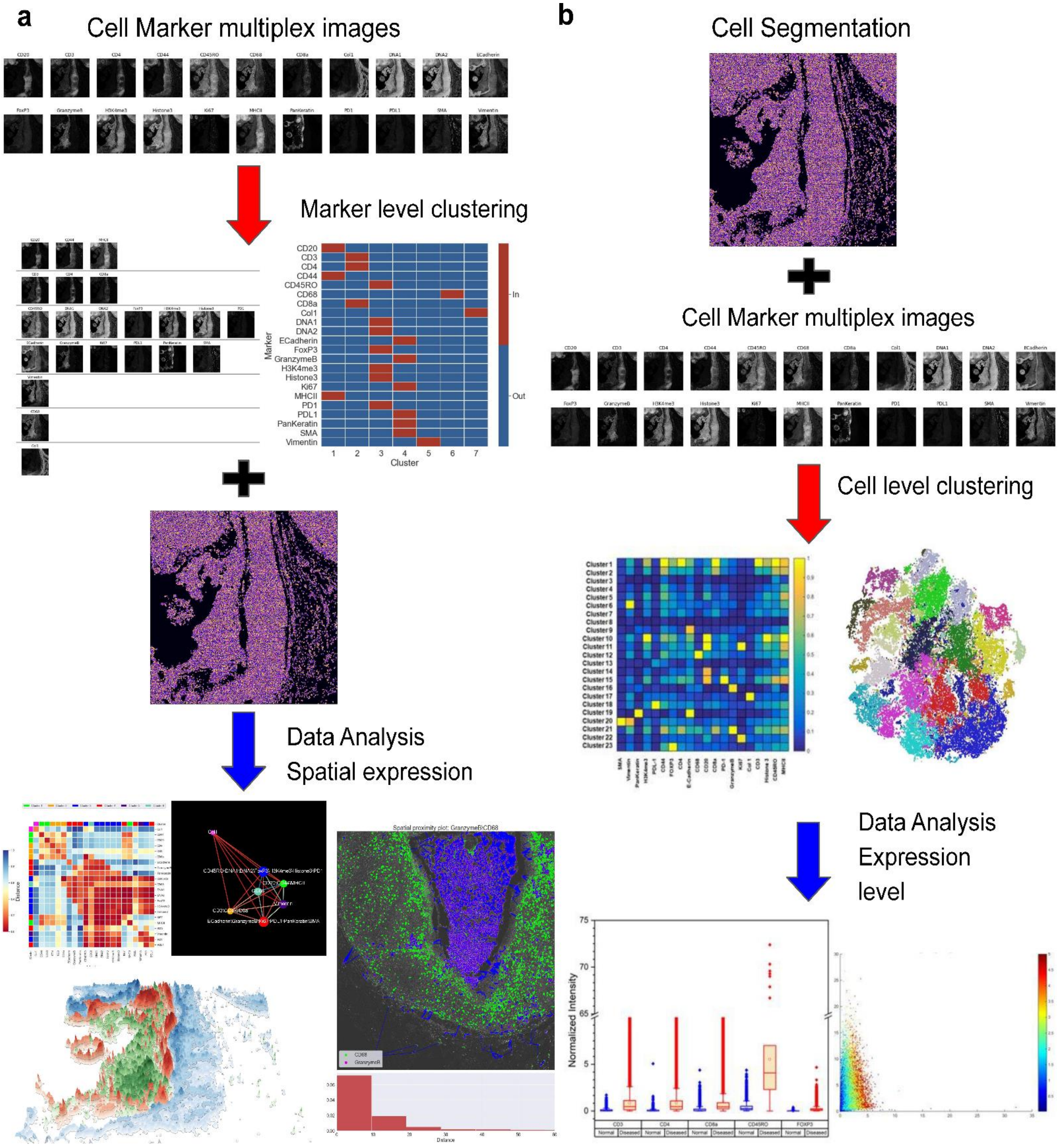
a
Cell Marker multiplex images
Marker level clustering
CD20
CD3
CD4
CD44
CD45RO
CD68
CD8a
Col1
DNA1
DNA2
ECadherin
FoxP3
GranzymeB
H3K4me3
Histone3
Ki67
MHCII
PD1
PDL1
PanKeratin
SMA
Vimentin
Marker
Cluster
In
Out
Data Analysis
Spatial expression
Spatial proximity plot: GranzymeB:CD68
Distance
b
Cell Segmentation
Cell Marker multiplex images
Cell level clustering
Data Analysis
Expression
level
Normalized Intensity
CD3
CD4
CD8a
CD45RO
FOXP3
Normal
Diseased

Supplementary Figure 22. SpatialViz combines top-down and bottom-up approaches.

Comparison of two mainstream analysis approaches in the right and left columns.

Both approaches utilize the same image dataset with cell segmentation and marker expression images. The bottom-up analysis processes the single-cell level information, and the top-down approach visualizes the marker anatomy.

a Top-down approach maps the multiple markers' anatomical features and then combines the single-cell level information from the bottom-up approach for a hybrid data analysis.

b Bottom-up approach starts with the single-cell segmentation information and quantifies each cell presence in the multiplexed images for quantification of cellular abundance and co-expressions.

Marker Name	Metal	Isotope	Antibody Concentration (mg/ml)	Final Dilution	Source	Catalog Number
**CD20**	Dy	161	0.5	1:400	Fluidigm	3161029D
**CD3**	Er	170	0.5	1:100	Fluidigm	3170019D
**CD4**	Gd	156	0.5	1:200	Fluidigm	3156033D
**CD45RO**	Yb	173	0.5	1:50	Fluidigm	3173016D
**CD68**	Tb	159	0.5	1:50	Fluidigm	3159035D
**CD8a**	Dy	162	0.5	1:100	Fluidigm	3162034D
**FoxP3**	Gd	155	0.5	1:30	Fluidigm	3155016D
**Pan-Keratin**	Nd	148	0.5	1:100	Fluidigm	3148020D
**Granzyme B**	Er	167	0.5	1:100	Fluidigm	3167021D
**Ki-67**	Er	168	0.5	1:50	Fluidigm	3168022D
**PD-1**	Ho	165	0.5	1:30	Fluidigm	3165039D
**PD-L1**	Nd	150	0.5	1:30	Fluidigm	3150031D
**Alpha-SMA**	Pr	141	0.5	1:200	Fluidigm	3141017D
**Collagen type 1**	Tm	169	0.5	1:300	Fluidigm	3169023D
**E-cadherin**	Gd	158	0.5	1:50	Fluidigm	3158029D
**Histone 3**	Yb	171	0.5	1:50	Fluidigm	3171022D
**Vimentin**	Nd	143	0.5	1:100	Fluidigm	3143027D
**CD44**	Eu	153	0.5	1:100	Fluidigm	3153029D
**MCHII**	Lu	175	1.64	1:200	Abcam	ab55152
**H3K9me3**	Sm	149	1.36	1:400	Abcam	ab232324
**Intercalator**	Ir	192/193	125uM stock	1:400	Fluidigm	201192A

Supplementary Table 1. The isotope-labeled antibody panel for imaging mass cytometry experiments.

The table shows all the markers investigated in this study alongside their conjugated metal isotope, antibody concentration, and dilution used in the final cocktail staining mix.

Supplementary Notes:

Details about the Fluidigm Imaging Mass Cytometer

ROIs size and Imaging Time:

Regions of interest (ROIs) chosen from the tonsil samples were approximately 2500 µm x 2500 µm. The laser type is Nd: YAG with a wavelength of 213-nm. The energy output was less than or equal to 3µJ. It takes 2 hours to image $1mm^2$; thereby, it took approximately 12.5 hours to acquire individual ROIs on the tonsil sections.

**Description**	**System Specifications**
Laser type	Nd: YAG
Wavelength	213 nm
Energy Output	3µJ
Tissue thickness for full ablation	≤ 7µm thickness
Sample size	≥ 15 mm x 45 mm
Scan Area	≥ 1 mm^2 / 2Hr (@200Hz)

Supplementary Table 2. System specification for the Hyperion imaging system by Fluidigm.

**Slide**	**Imaging Time (H: M:S)**	**Laser Power (dB)**	**Ablation Power (Hz)**
Normal Tonsil 1	9:59:51	2.5	200
Normal Tonsil 2	9:59:51	2.5	200
Normal Tonsil 3	9:59:51	2	200
Diseased Tonsil 1	7:45:34	2	200
Diseased Tonsil 2	9:59:51	2	200
Diseased Tonsil 3	9:59:51	2.5	200

Supplementary Table 3. Experimental details regarding samples, imaging time, laser power, and ablation power were used to obtain the paper's data.

Since these product specifications can vary between different experimental runs, we also included some of the parameters associated with two experimental runs for one normal tonsil sample and one diseased tonsil sample. As shown in the snapshots below, different ablation energy values were tried out before choosing the ROIs' final values. This optimization process was done by testing out different values of ablation energies on smaller regions 100 µm x 100 µm instead of 2500 µm x 2500 µm. The value that resulted in a strong signal with the minimum background was finally chosen for the bigger ROIs.

Fluidigm Hyperion imaging system machine details for all ROIs used to obtain the data

Normal Tonsil 1

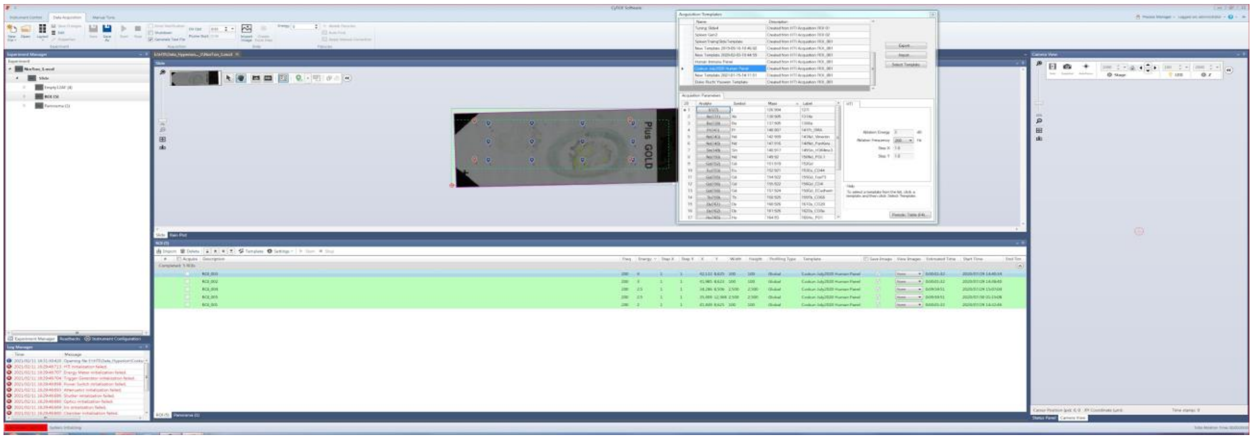

Normal Tonsil 2

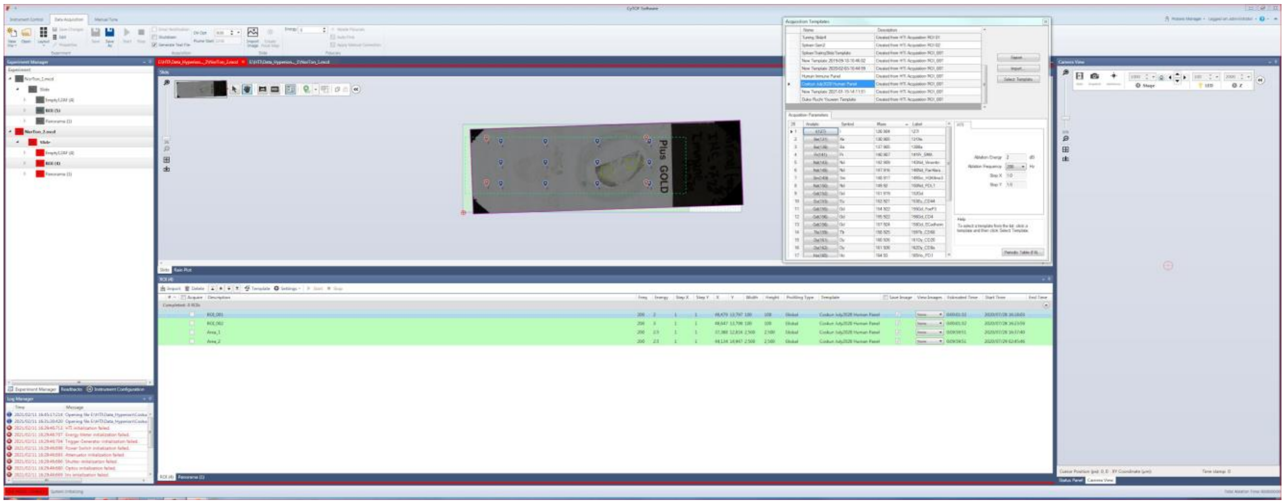

Normal Tonsil 3

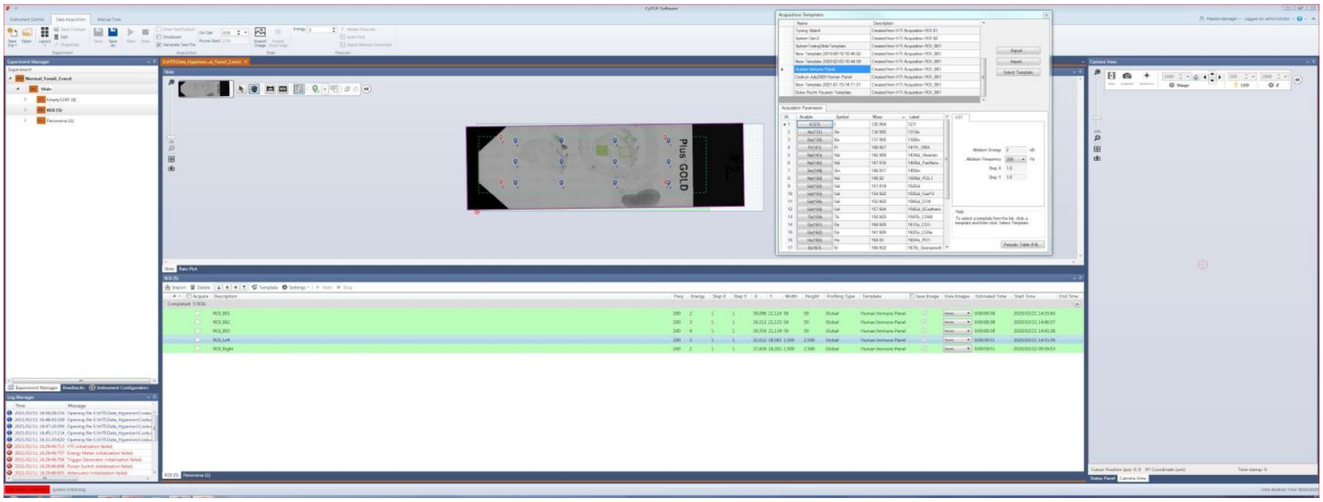

Diseased Tonsil 1

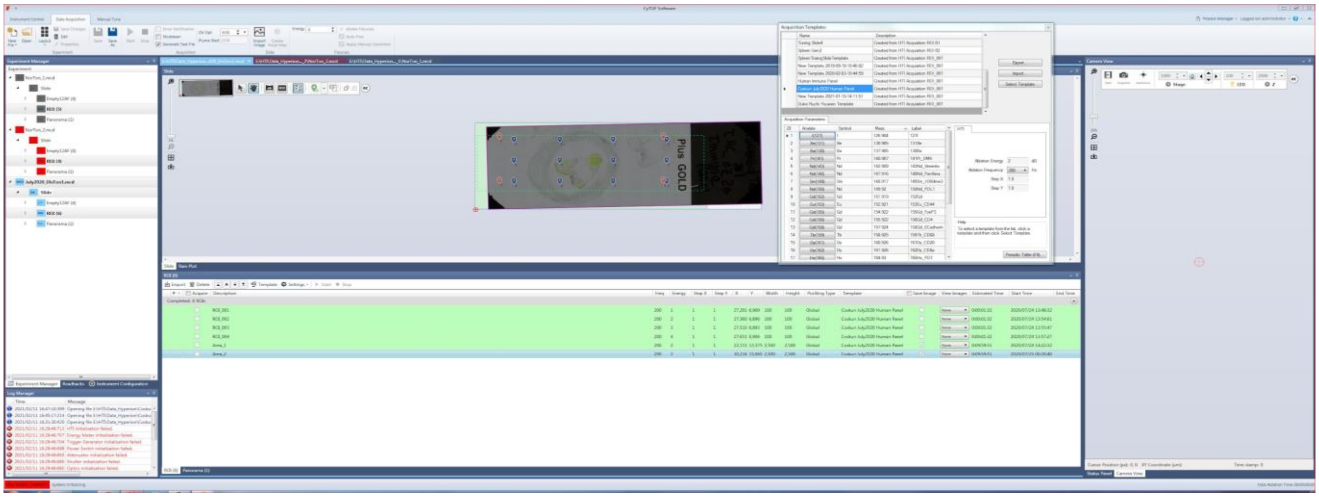

Diseased Tonsil 2

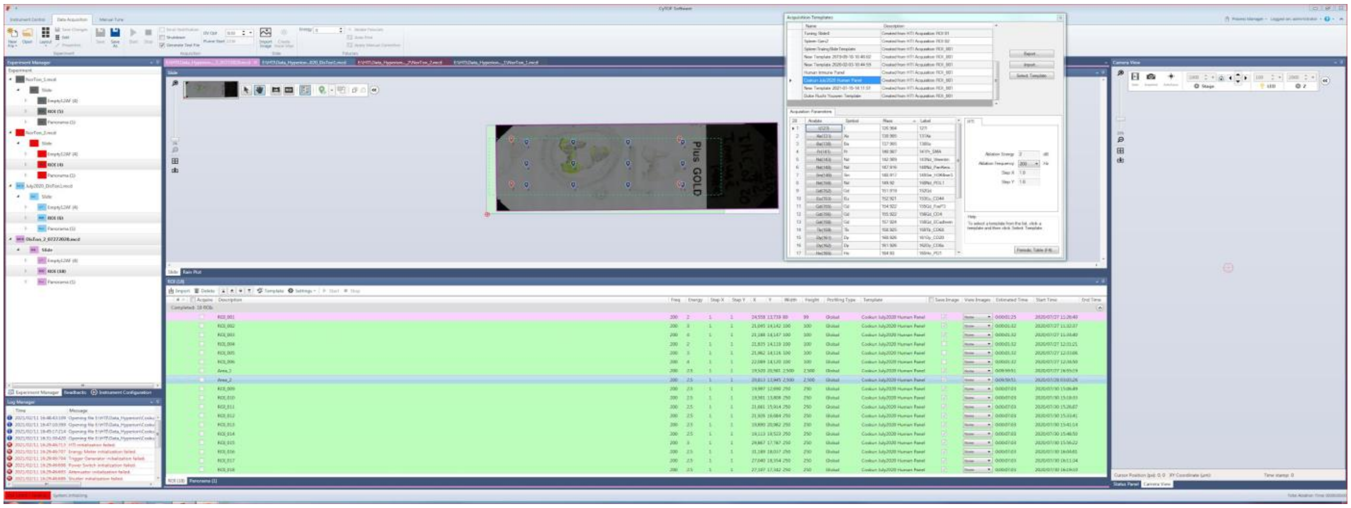

Diseased Tonsil 3

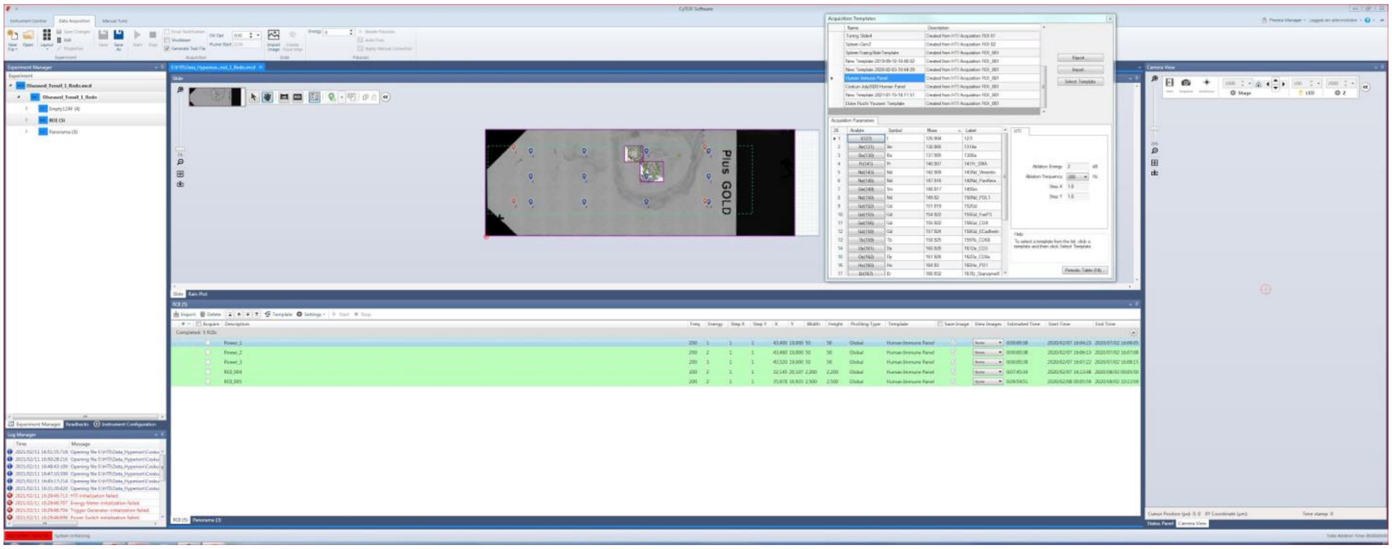

Open-source Software Notes

The following open-source software was used in the SpatialViz image processing, analysis, and visualization pipelines:

1. SciPy[1]: http://www.scipy.org
2. Numpy[2]: http://www.numpy.org
3. Matplotlib[3]: http://www.matplotlib.org
4. Plotly[4]: https://plotly.com/python/
5. Scikit-learn[5]: http://scikit-learn.org/stable/index.html
6. Pandas[6]: https://pandas.pydata.org/
7. Scikit-image[7]: http://scikit-image.org/
8. OpenCV[8]: https://opencv.org/
9. ImageJ[9]: https://imagej.nih.gov/ij/
10. CellProfiler[10]: https://cellprofiler.org/
11. Seaborn[11]: http://seaborn.pydata.org
12. NetworkX[12]: https://networkx.org/

References:

1. Jones E., Oliphant E., Peterson P., et al. SciPy: Open Source Scientific Tools for Python, (2001-).
2. Oliphant E. A., Guide to NumPy, Trelgol Publishing (2006)
3. Hunter J. D. Matplotlib: A 2D graphics environment, Computing In Science & Engineering (2007).
4. Sievert, Carson. Interactive Web-Based Data Visualization with R, Plotly, and shiny. CRC Press, (2020).
5. Pedregosa et al., Scikit-learn: Machine learning in Python, Journal of Machine Learning Research 12, 2825-2830 (2011).
6. McKinney W. Data Structures for Statistical Computing in Python. Proceedings of the 9th Python in Science Conference, 51-56 (2010).
7. Van der Walt S. et al., scikit-image: Image processing in Python. PeerJ 2:e453 (2014).
8. Bradski G., The OpenCV Library. Dr. Dobb's Journal of Software Tools (2000).
9. Ruden C.T., et al. ImageJ2: ImageJ for the next generation of scientific image data. BMC Bioinformatics 18:529 (2017).
10. McQuin, C. et al. CellProfiler 3.0: Next-generation image processing for biology. PLOS Biol. 16, e2005970 (2018).
11. Waskom, M. L. Seaborn: statistical data visualization. Journal of Open Source Software, 6(60), 3021, (2021).
12. Hagberg, A. A., Schult, D. A. & Swart, P. J. Exploring Network Structure, Dynamics, and Function using NetworkX. in Proceedings of the 7th Python in Science Conference (eds. Varoquaux, G., Vaught, T. & Millman, J.) 11–15 (2008).
