# Supplementary material for: Spatially visualized single-cell pathology of highly multiplexed protein profiles in health and disease
Source: Commun Biol. 2021 May 27;4:632. doi: 10.1038/s42003-021-02166-2 (PMC8160218; doi:10.1038/s42003-021-02166-2)
Supplement: Supplementary file 3 — Descriptions of Additional Supplementary Files [file 42003_2021_2166_MOESM3_ESM.pdf]

Descriptions of Additional Supplementary Files

Supplementary Data 1
Description: The csv files used in figure 2g-h and 3b-e.

Supplementary Movie 1
Description: Movie shows the 3D visualization of the topographic map composed of Granzyme B, CD44, and pankeratin for sample DT2.

Supplementary Movie 2
Description: Movie shows the 3D visualization of the topographic map composed of Granzyme B, CD44, and pankeratin for sample DT3.

Supplementary Movie 3
Description: Movie shows the 3D visualization of the topographic map composed of Granzyme B, CD44, and pankeratin for sample NT1.

Supplementary Movie 4
Description: Movie shows the 3D visualization of the topographic map composed of Granzyme B, CD44, and pankeratin for sample NT3.
